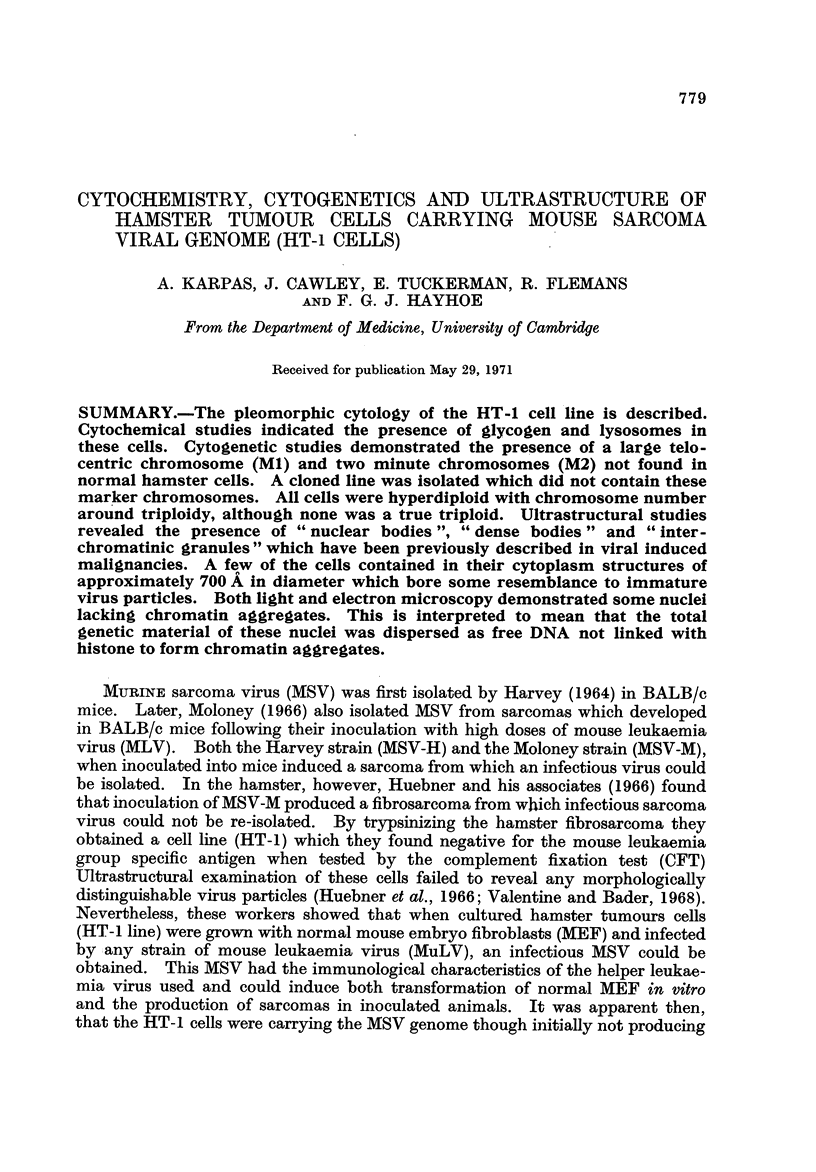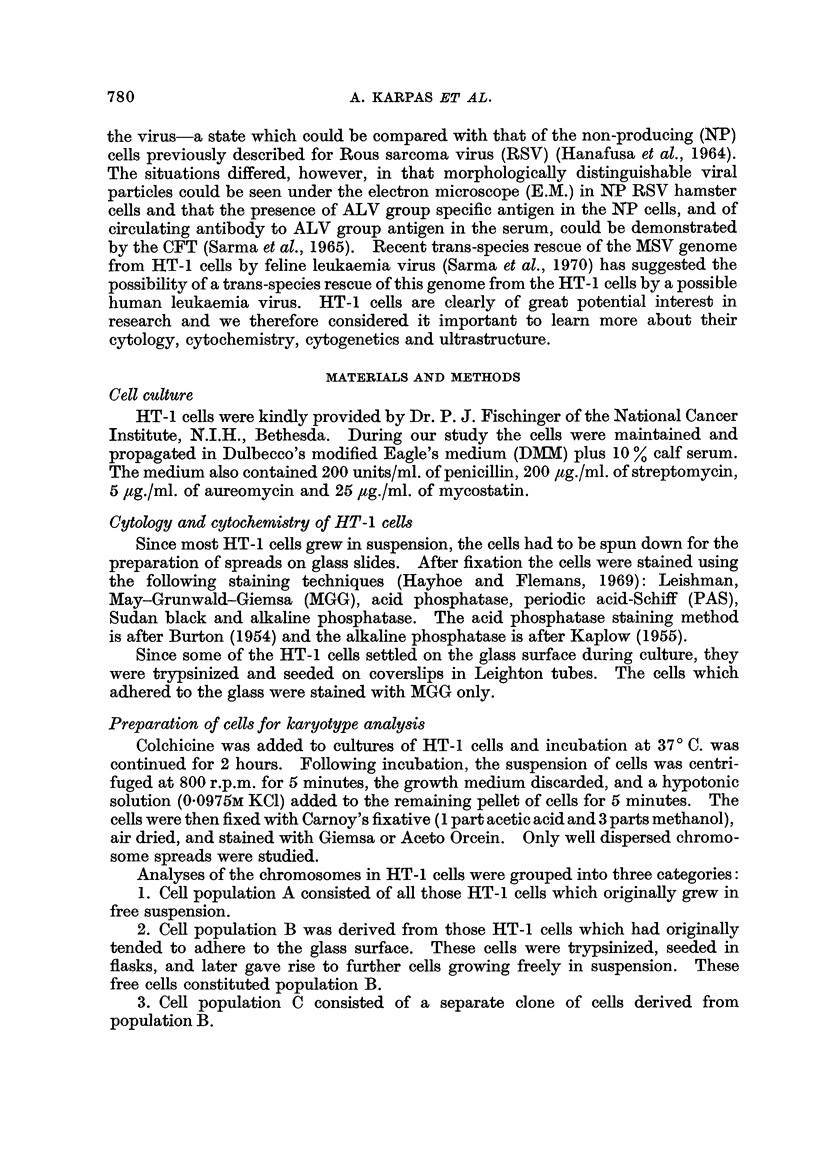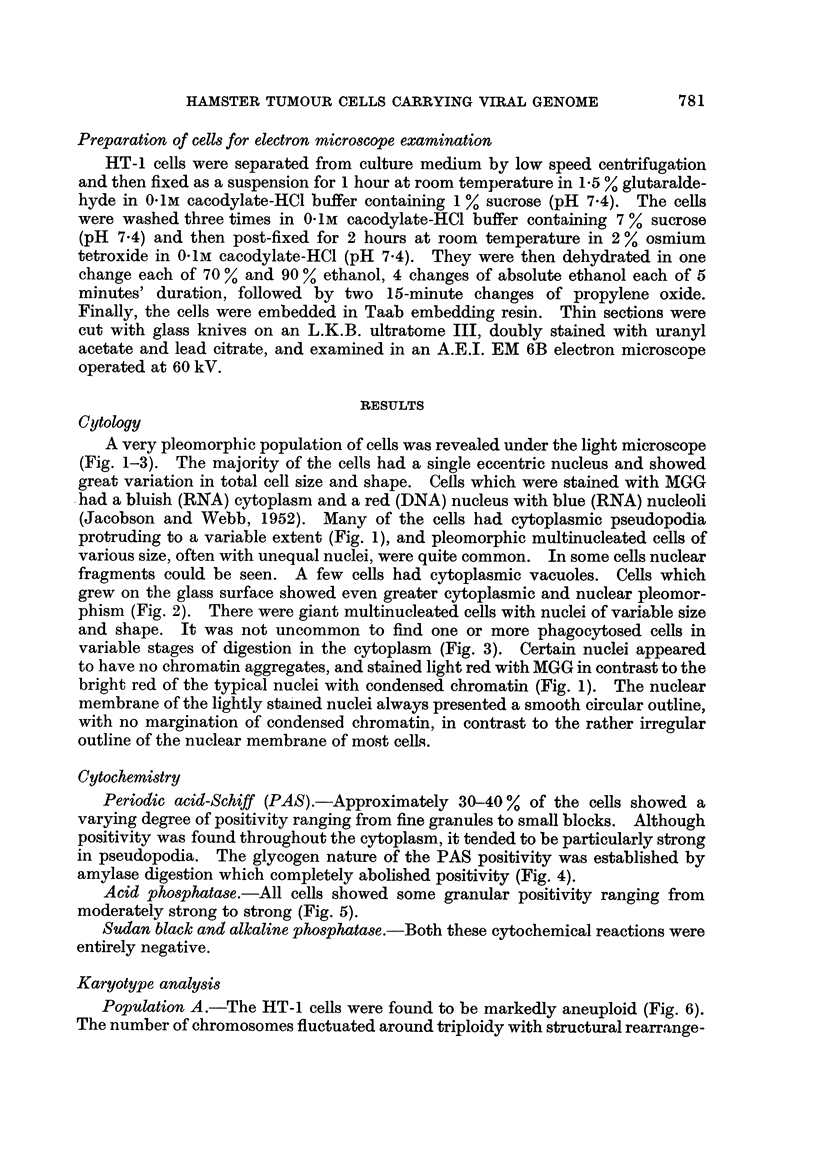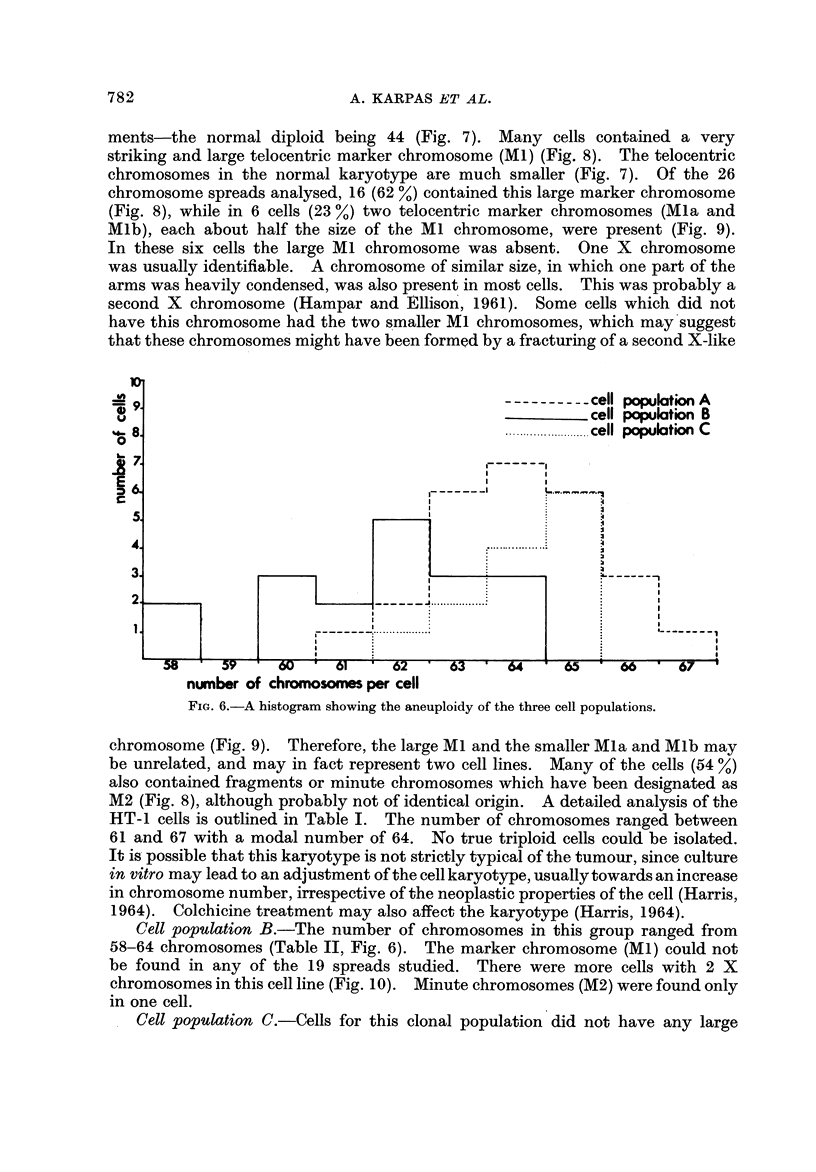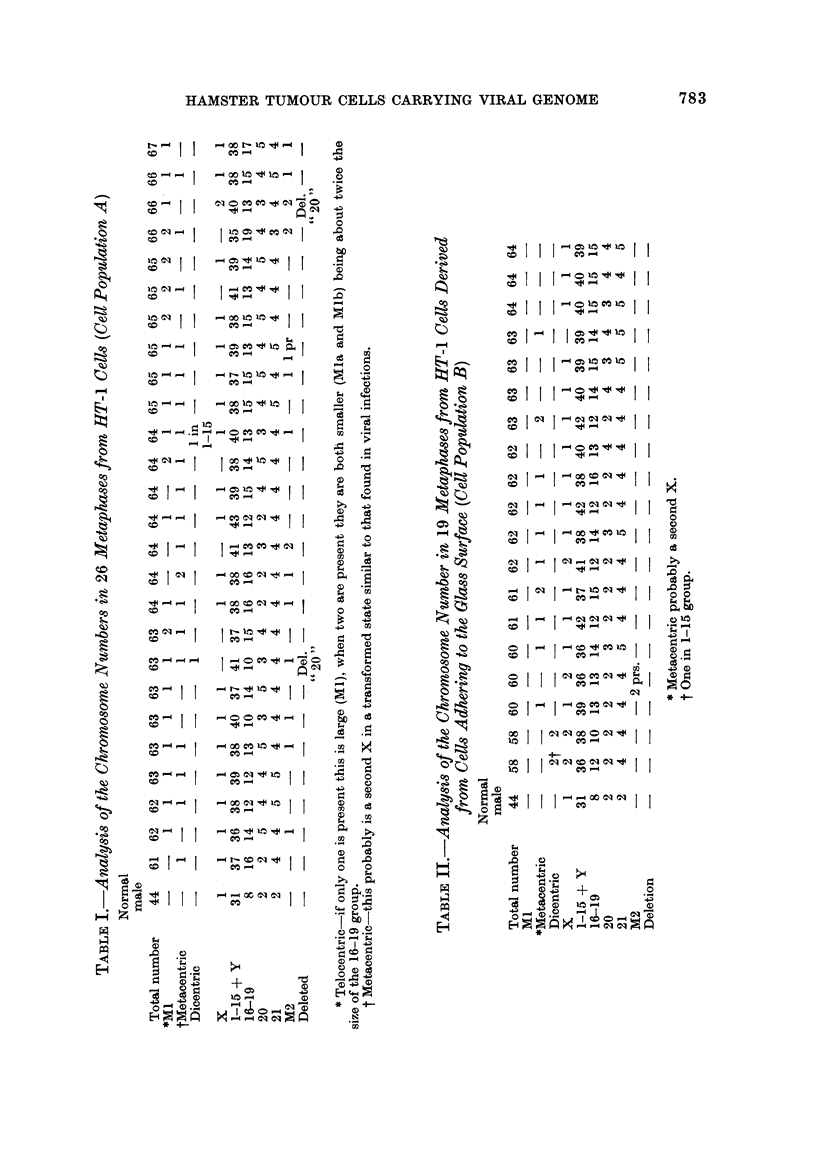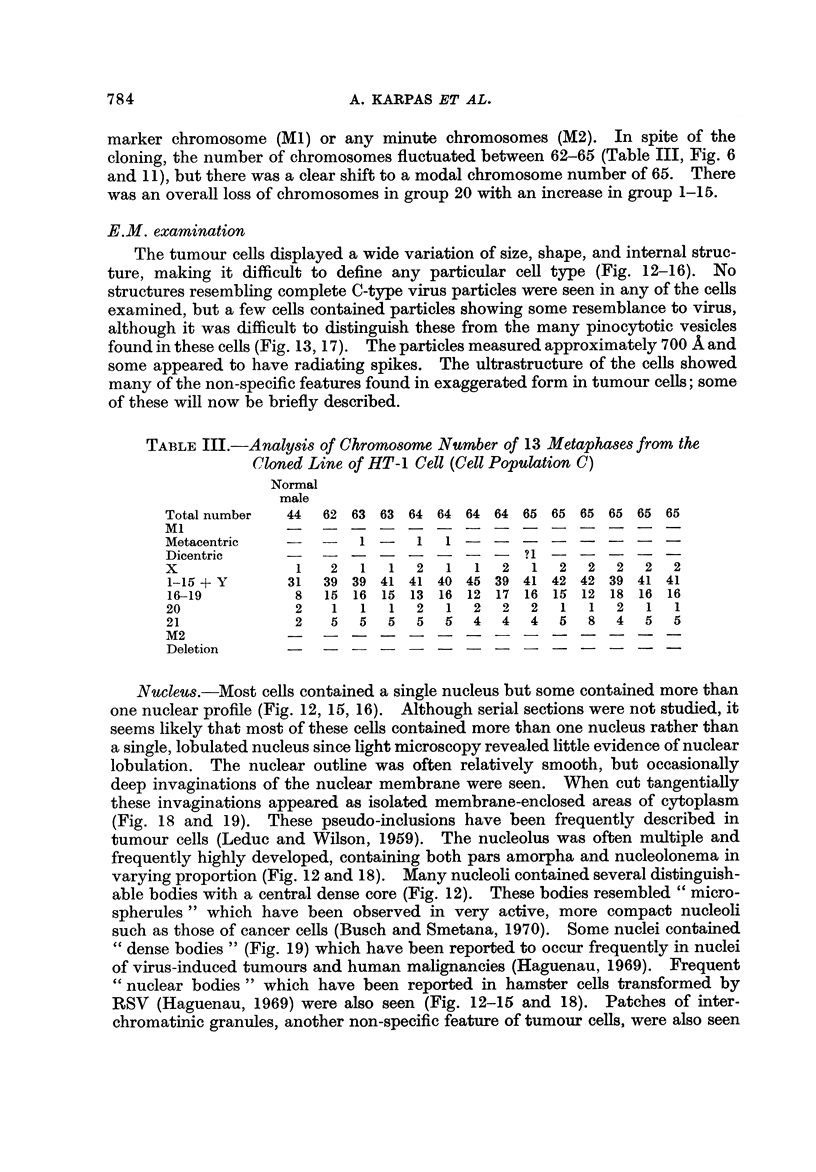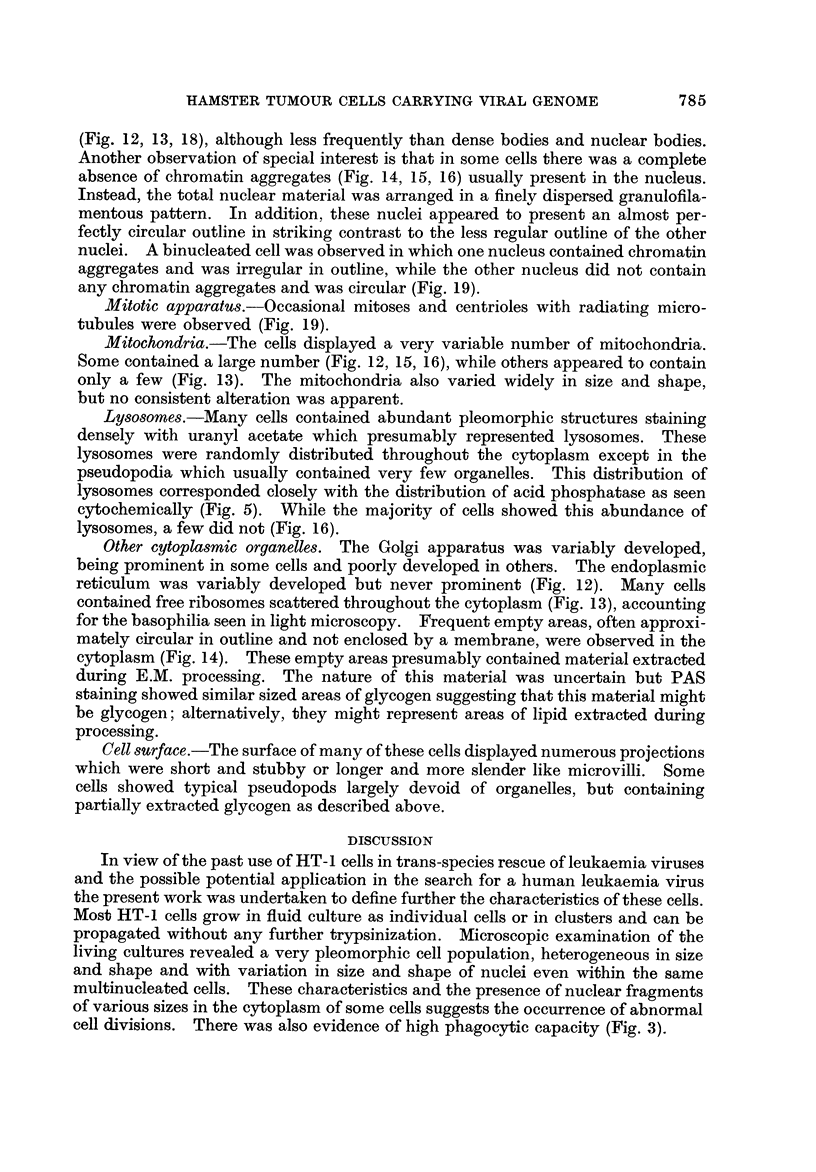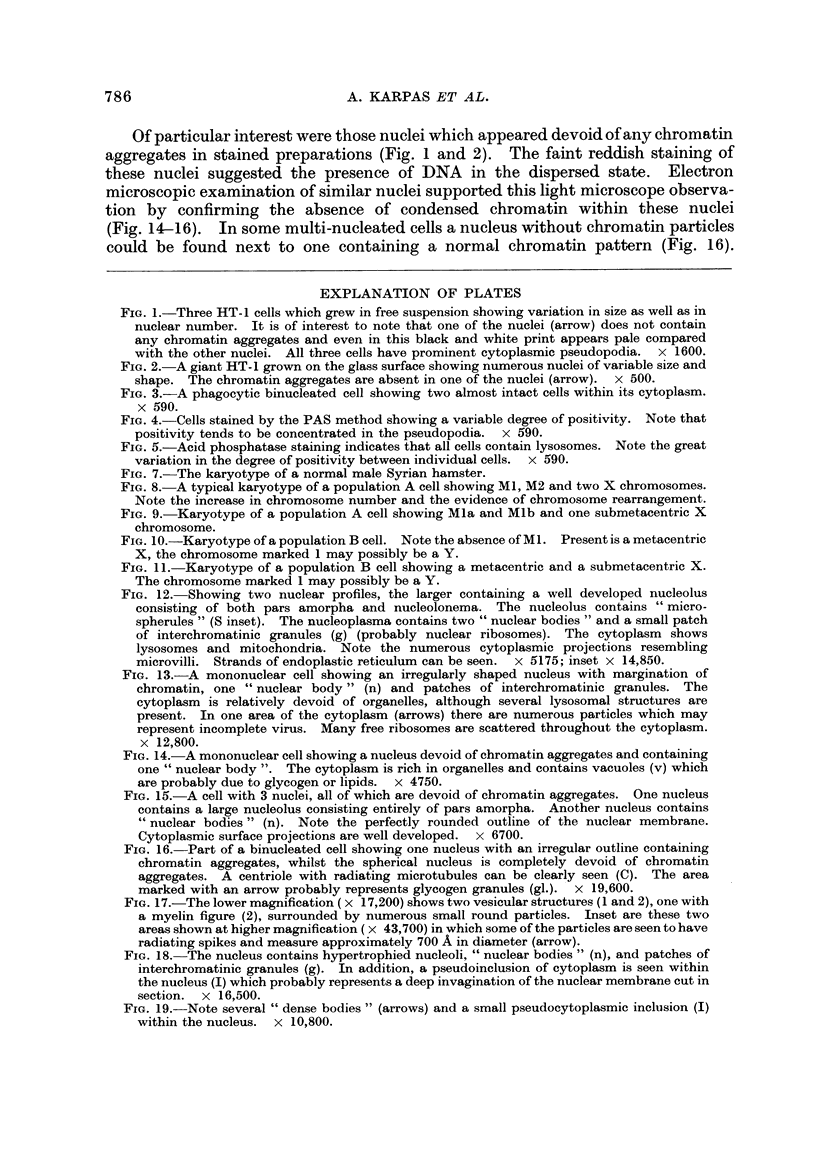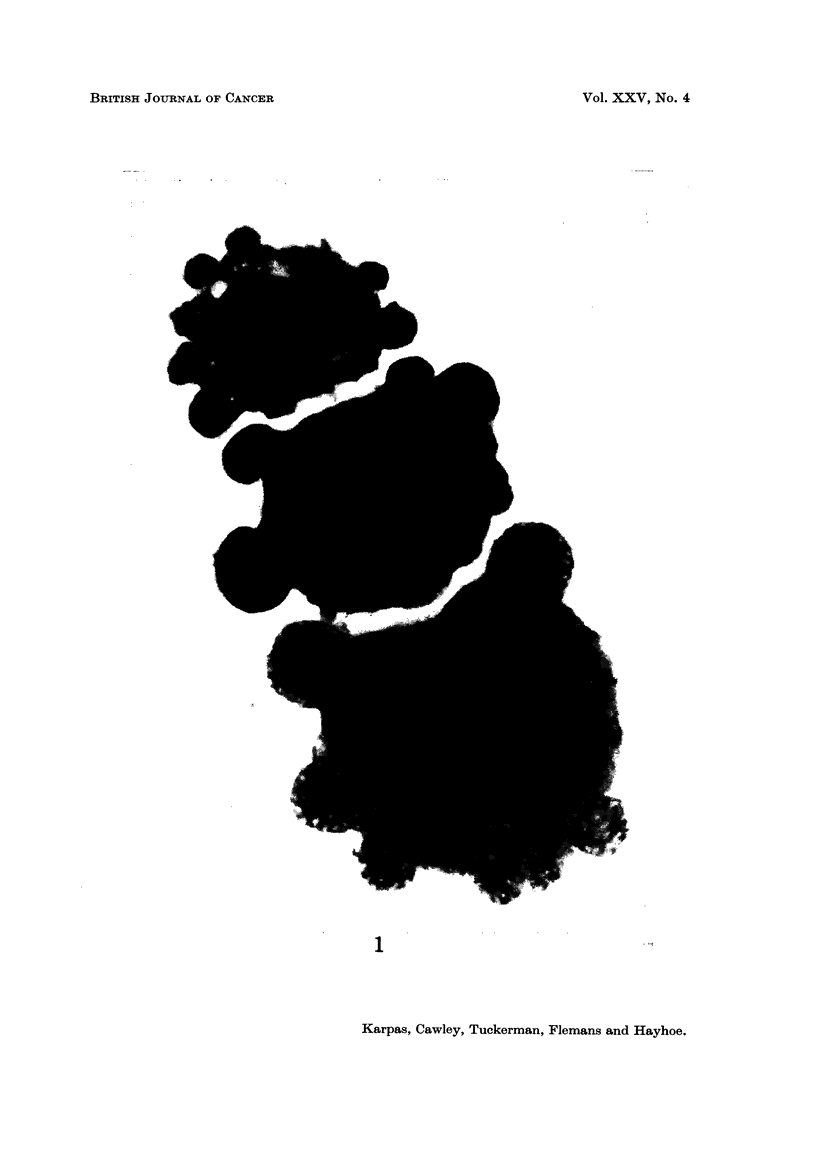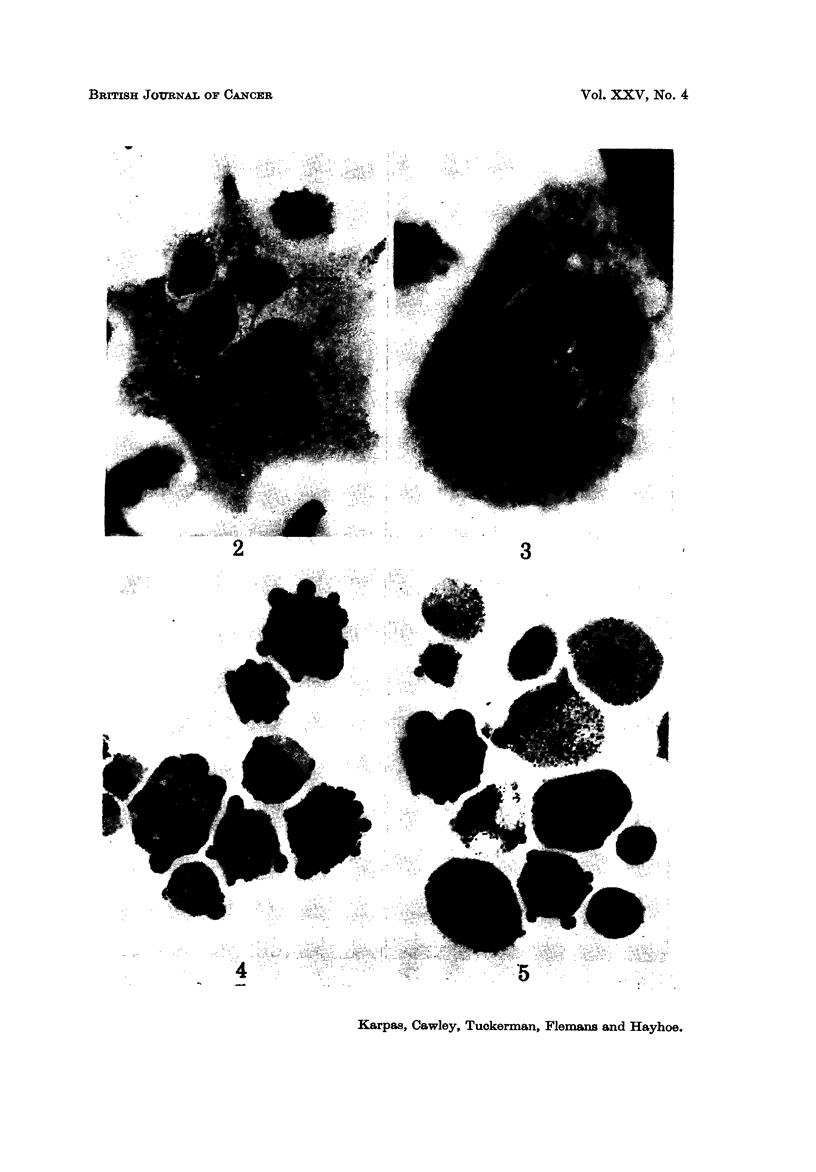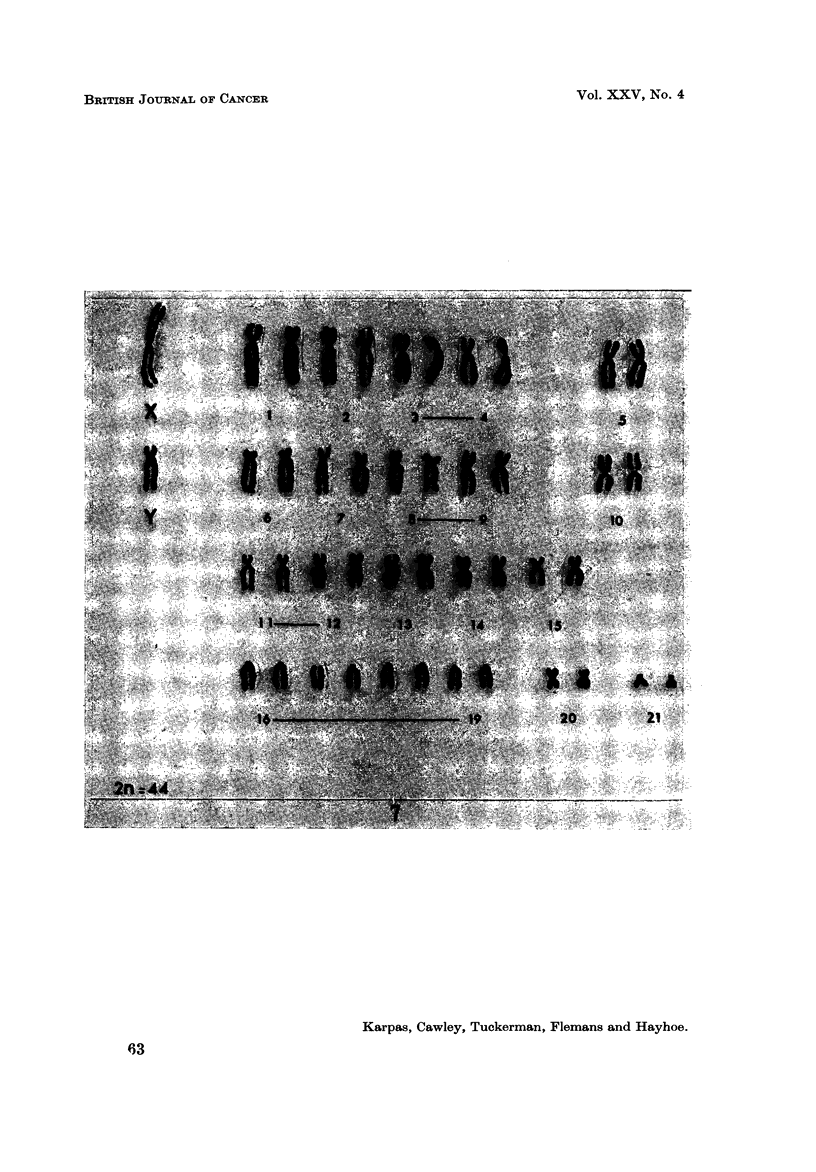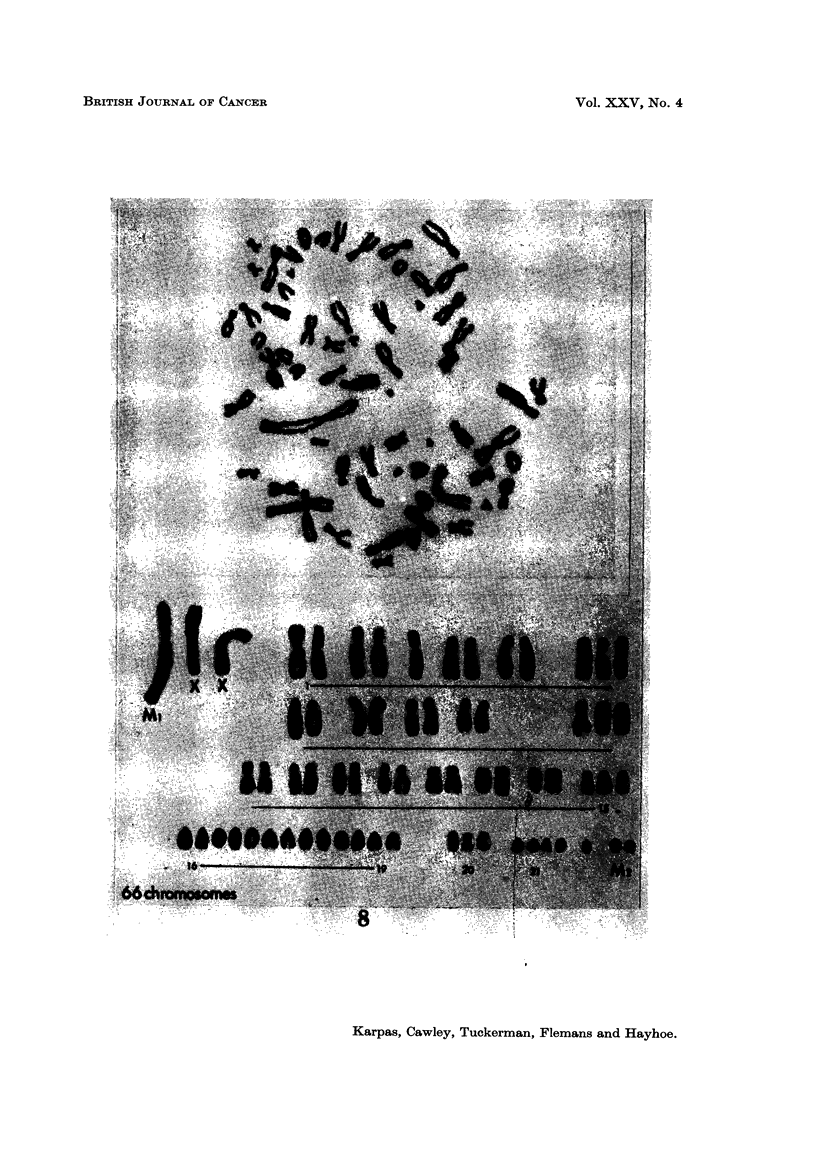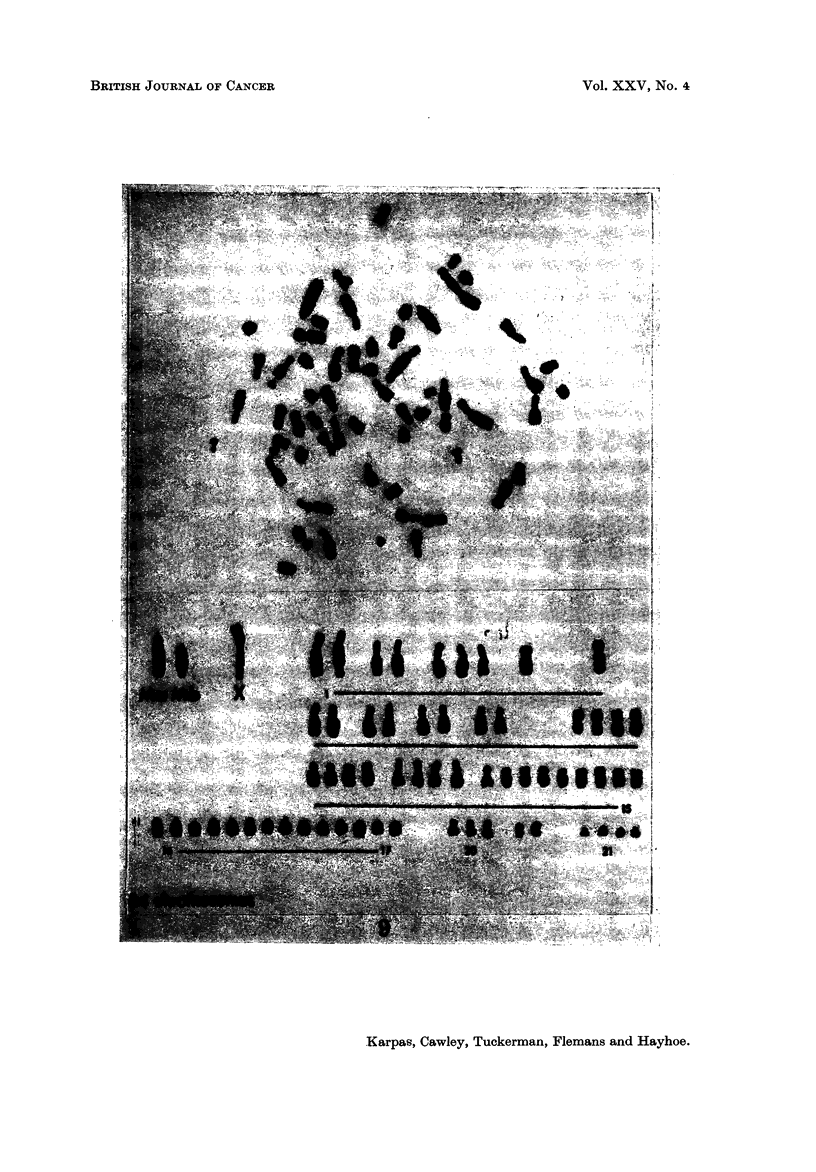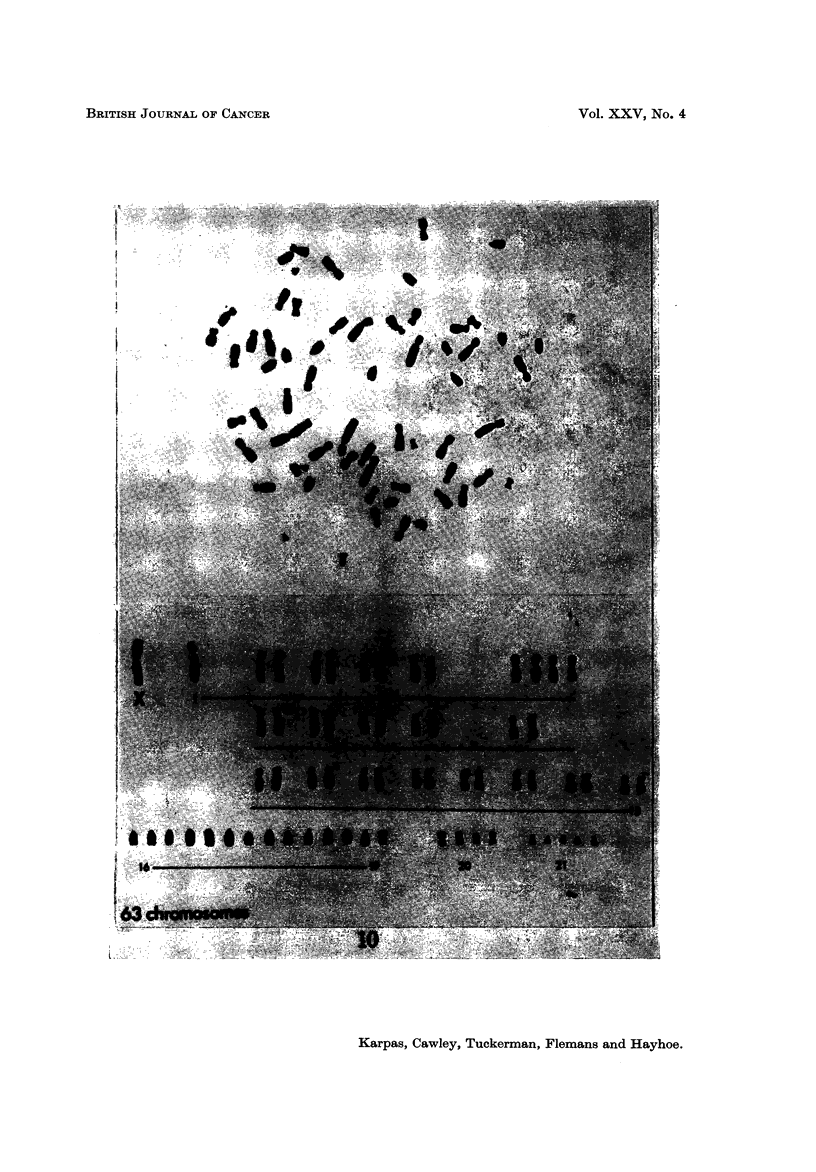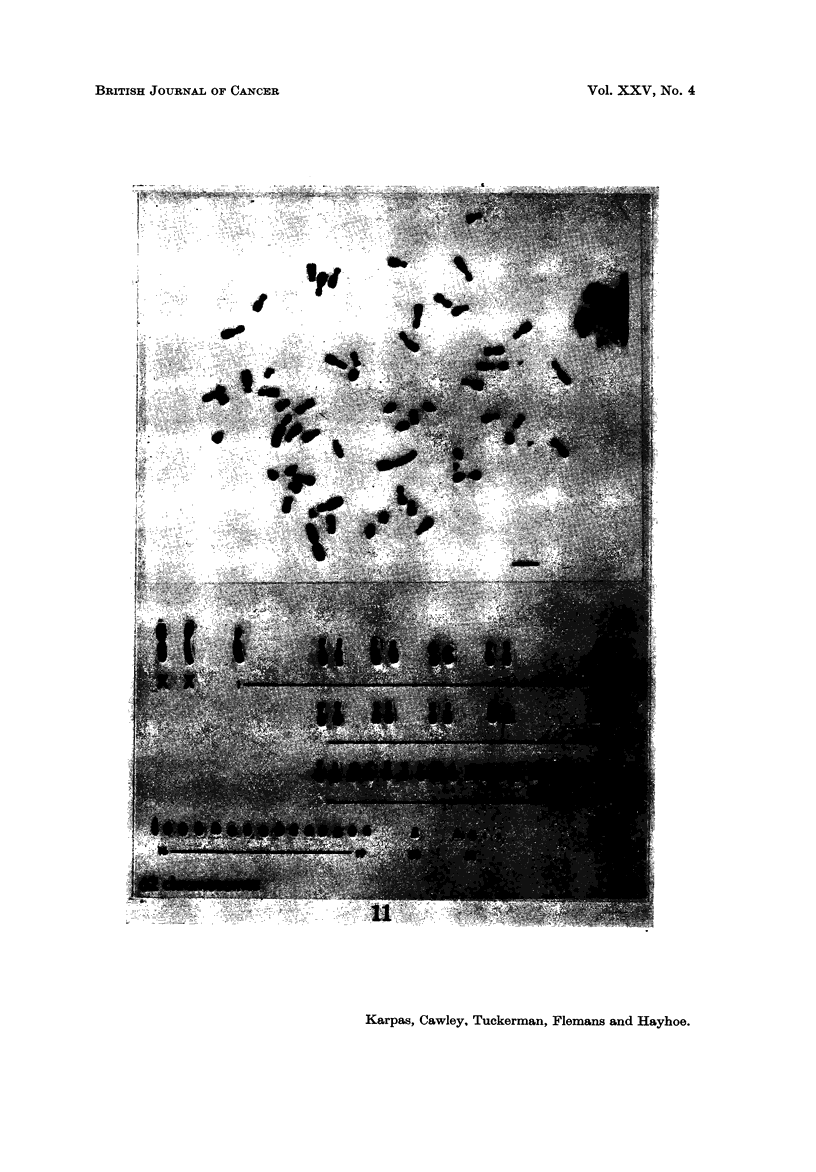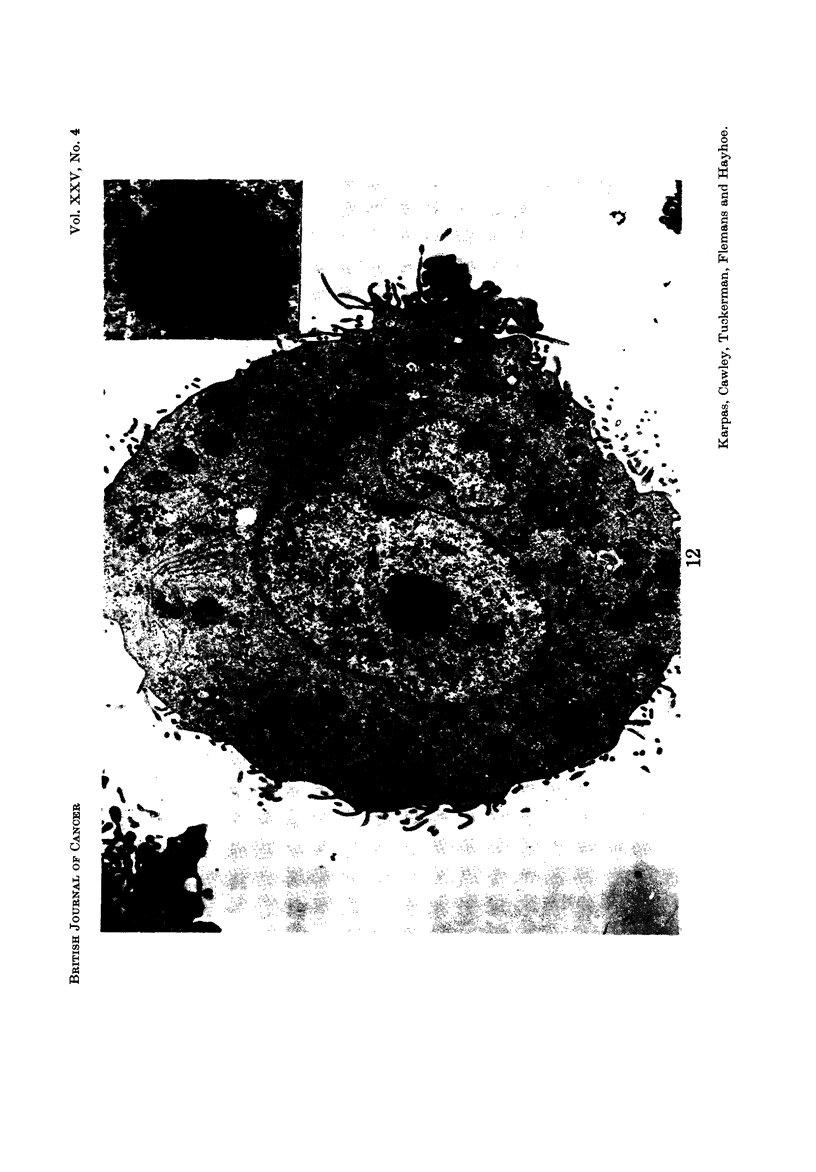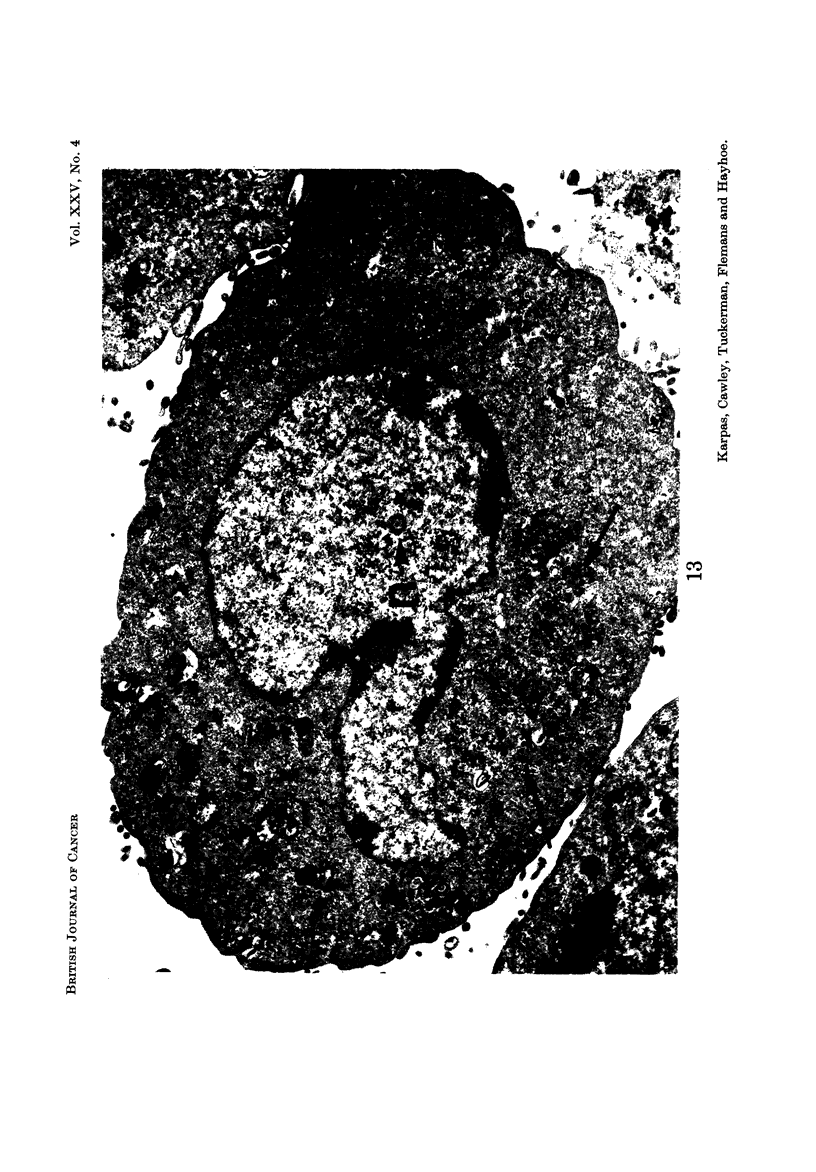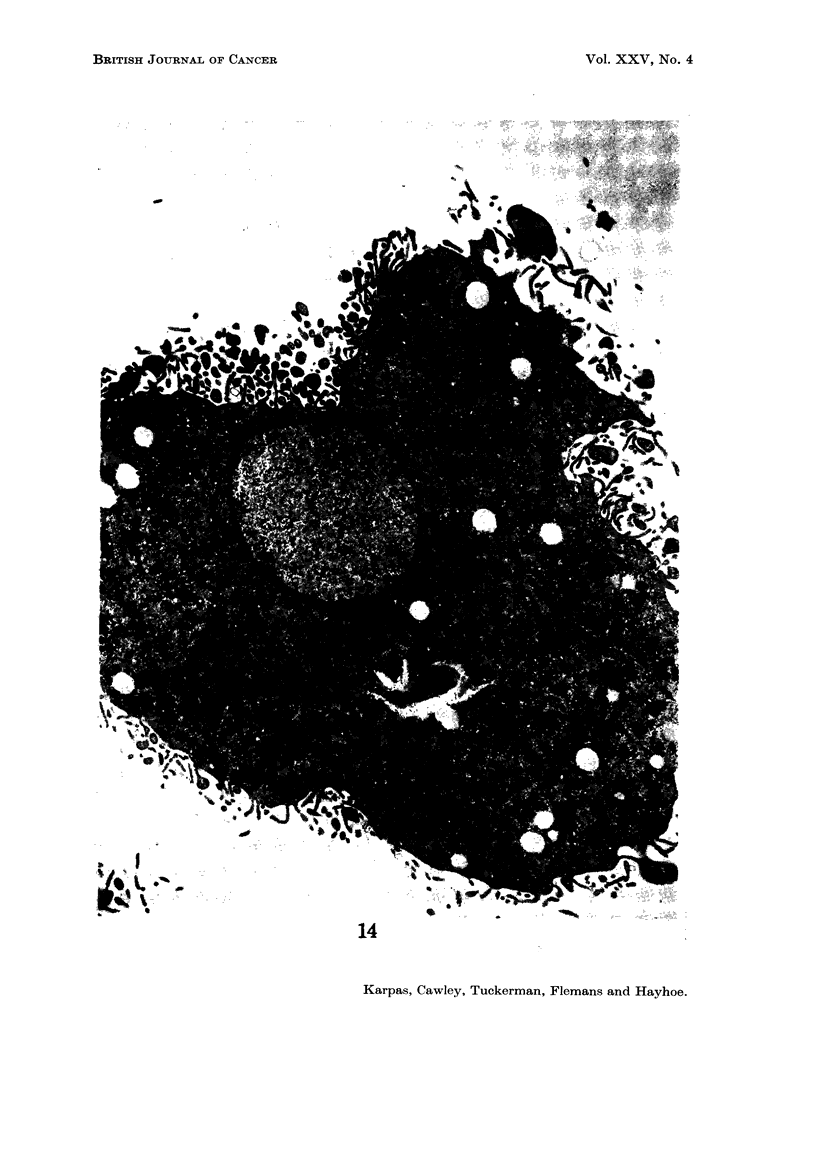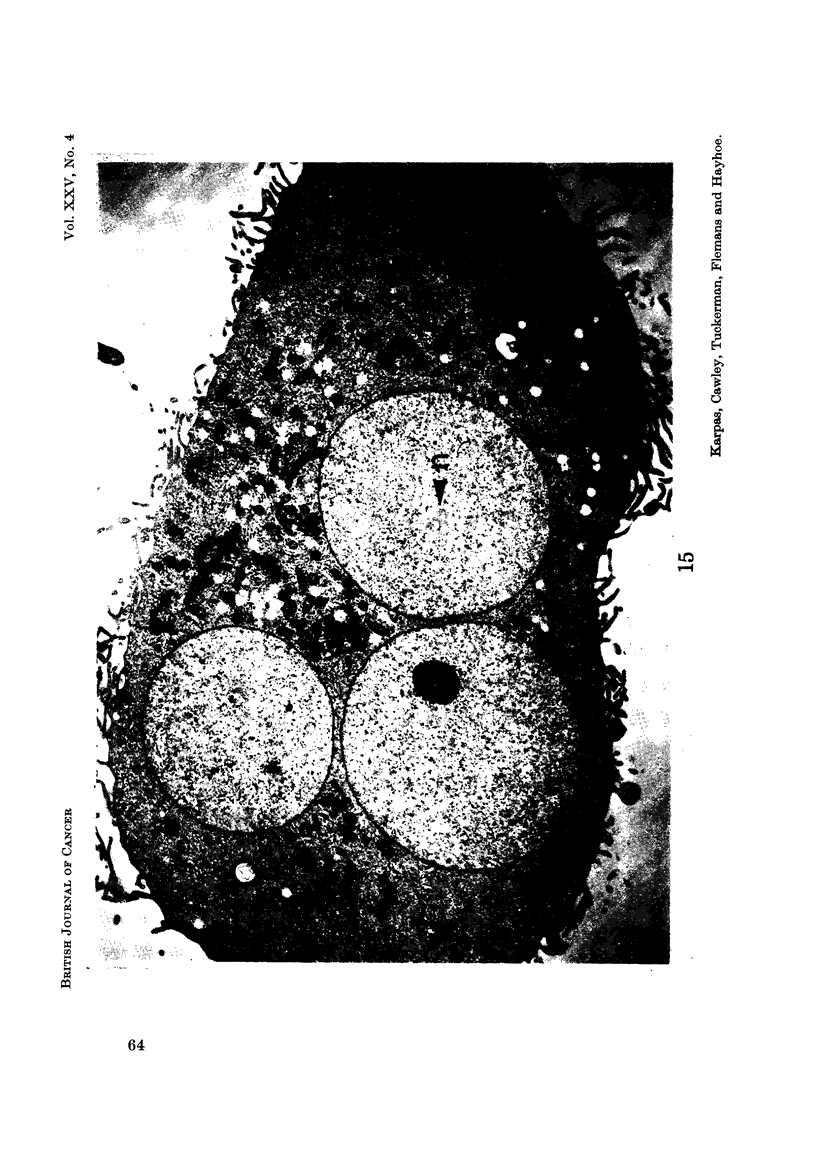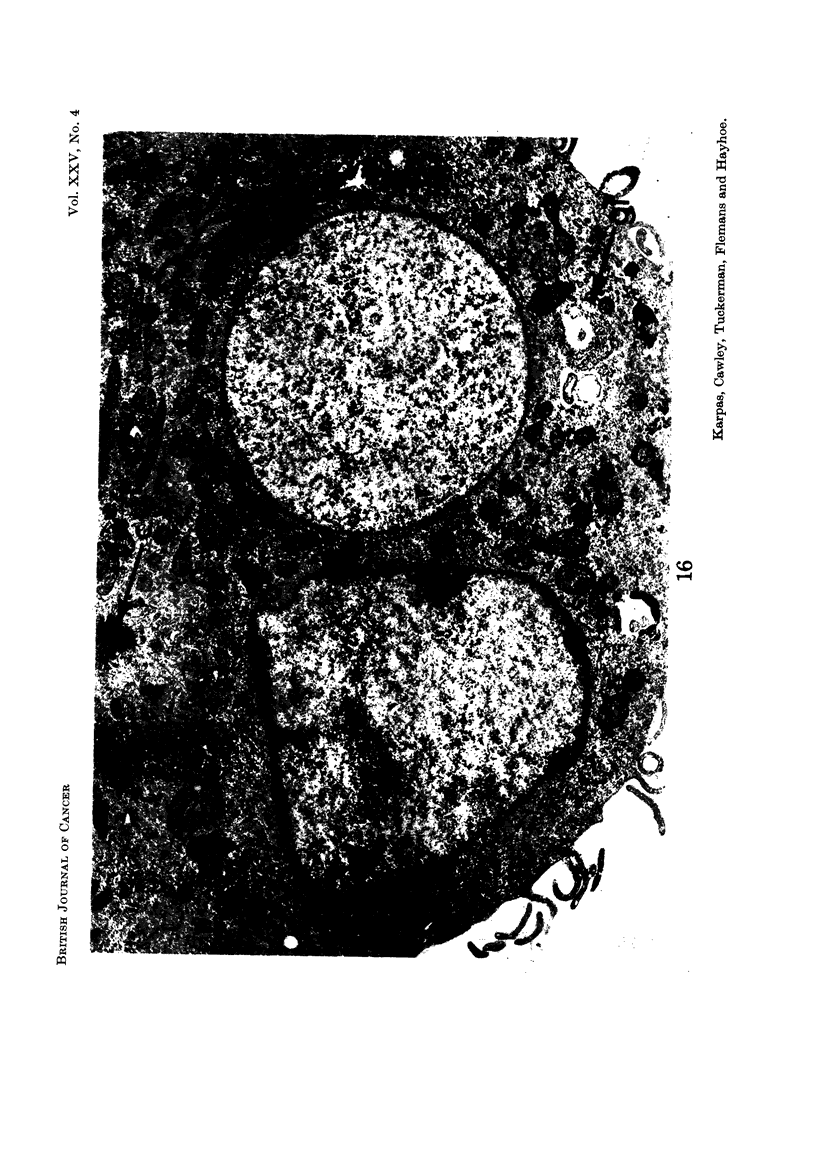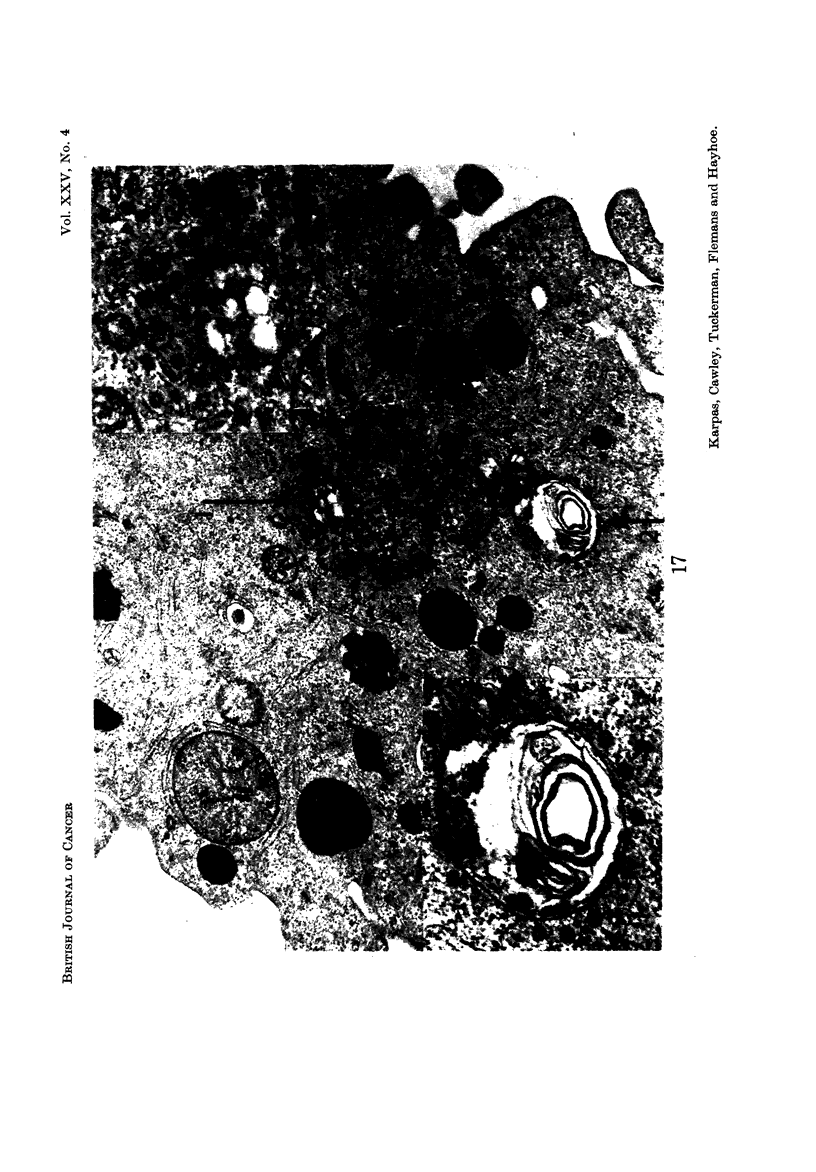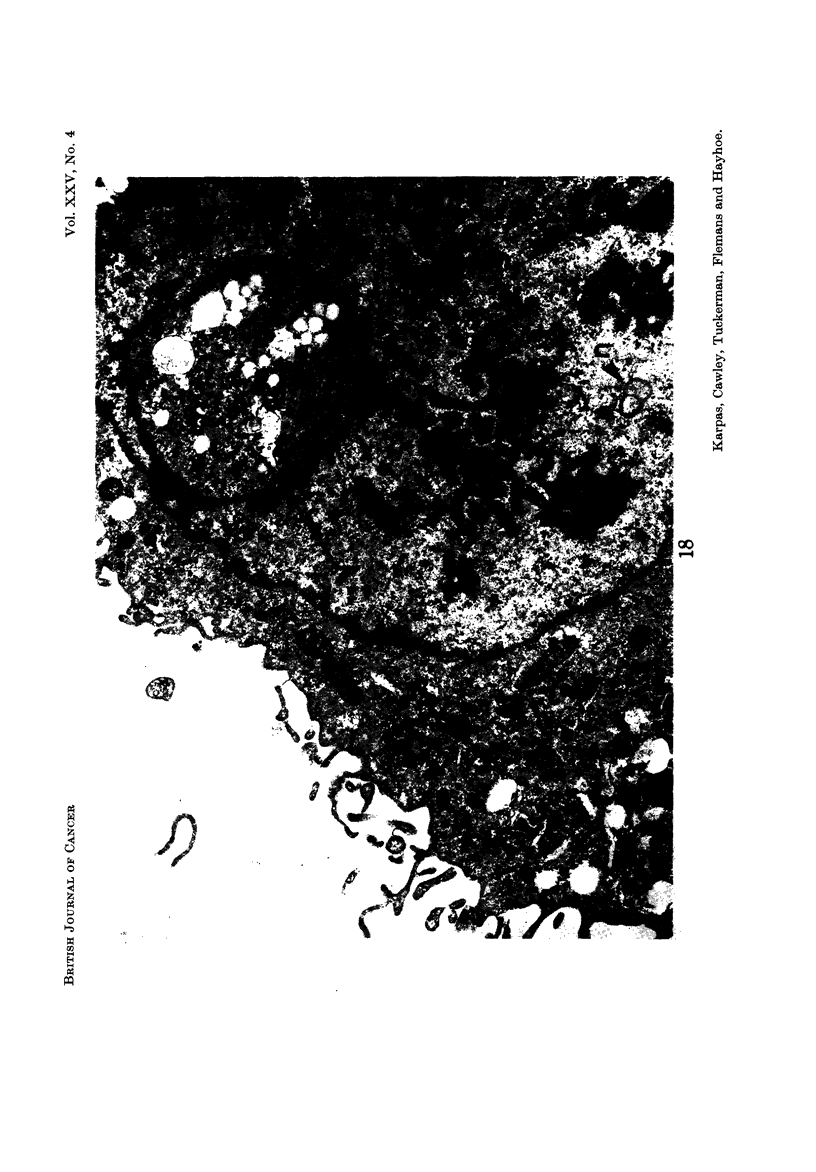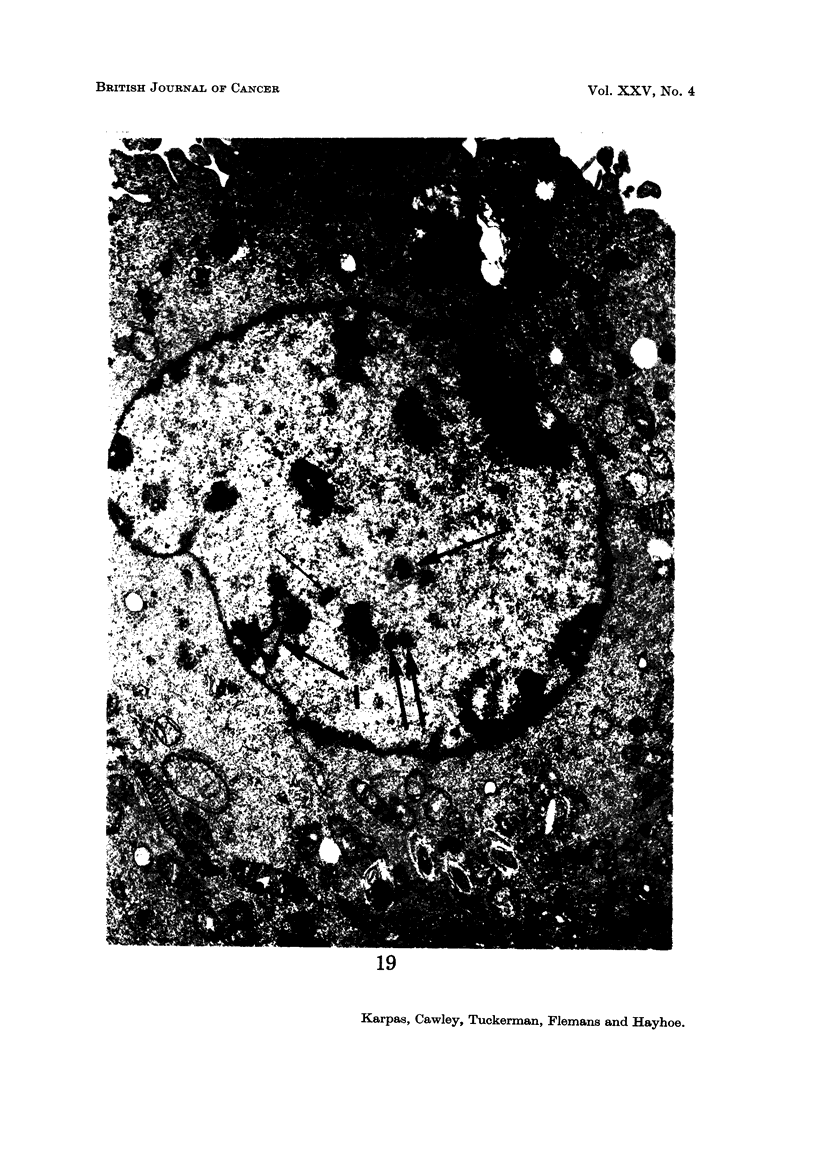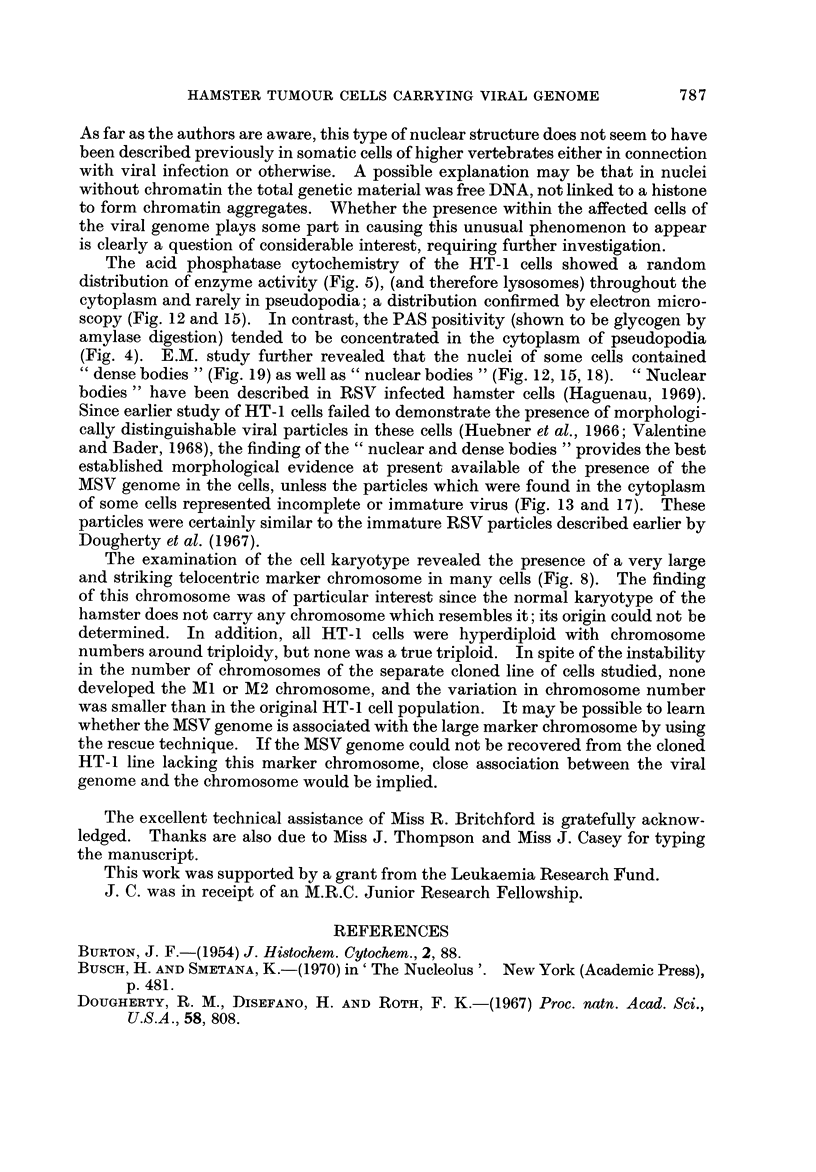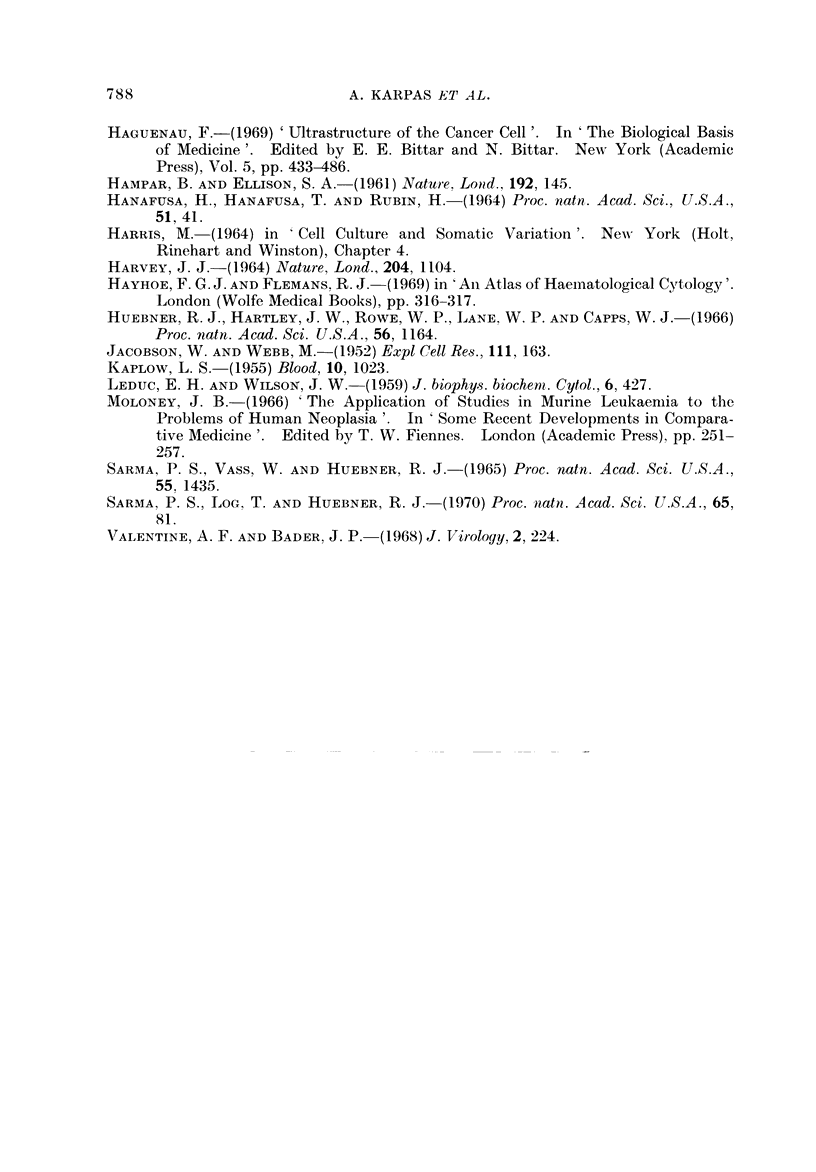# Cytochemistry, Cytogenetics and Ultrastructure of Hamster Tumour Cells Carrying Mouse Sarcoma Viral Genome (HT-1 Cells)

**DOI:** 10.1038/bjc.1971.92

**Published:** 1971-12

**Authors:** A. Karpas, J. Cawley, E. Tuckerman, R. Flemans, F. G. J. Hayhoe

## Abstract

**Images:**


### CYTOCHEMISTRY, CYTOGENETICS AND ULTRASTRUCTURE OF HAMSTER TUMOUR CELLS CARRYING MOUSE SARCOMA VIRAL GENOME (HT-1 CELLS)

A. KARPAS, J. CAWLEY, E. TUCKERMAN, R. FLEMANS AND F. G. J. HAYHOE

*From the Department of Medicine, University of Cambridge*

Received for publication May 29, 1971

**SUMMARY.—The pleomorphic cytology of the HT-1 cell line is described. Cytochemical studies indicated the presence of glycogen and lysosomes in these cells. Cytogenetic studies demonstrated the presence of a large telocentric chromosome (M1) and two minute chromosomes (M2) not found in normal hamster cells. A cloned line was isolated which did not contain these marker chromosomes. All cells were hyperdiploid with chromosome number around triploidy, although none was a true triploid. Ultrastructural studies revealed the presence of " nuclear bodies ", " dense bodies " and " interchromatinic granules " which have been previously described in viral induced malignancies. A few of the cells contained in their cytoplasm structures of approximately 700 Å in diameter which bore some resemblance to immature virus particles. Both light and electron microscopy demonstrated some nuclei lacking chromatin aggregates. This is interpreted to mean that the total genetic material of these nuclei was dispersed as free DNA not linked with histone to form chromatin aggregates.**

MURINE sarcoma virus (MSV) was first isolated by Harvey (1964) in BALB/c mice. Later, Moloney (1966) also isolated MSV from sarcomas which developed in BALB/c mice following their inoculation with high doses of mouse leukaemia virus (MLV). Both the Harvey strain (MSV-H) and the Moloney strain (MSV-M), when inoculated into mice induced a sarcoma from which an infectious virus could be isolated. In the hamster, however, Huebner and his associates (1966) found that inoculation of MSV-M produced a fibrosarcoma from which infectious sarcoma virus could not be re-isolated. By trypsinizing the hamster fibrosarcoma they obtained a cell line (HT-1) which they found negative for the mouse leukaemia group specific antigen when tested by the complement fixation test (CFT) Ultrastructural examination of these cells failed to reveal any morphologically distinguishable virus particles (Huebner *et al.*, 1966; Valentine and Bader, 1968). Nevertheless, these workers showed that when cultured hamster tumours cells (HT-1 line) were grown with normal mouse embryo fibroblasts (MEF) and infected by any strain of mouse leukaemia virus (MuLV), an infectious MSV could be obtained. This MSV had the immunological characteristics of the helper leukaemia virus used and could induce both transformation of normal MEF *in vitro* and the production of sarcomas in inoculated animals. It was apparent then, that the HT-1 cells were carrying the MSV genome though initially not producing the virus—a state which could be compared with that of the non-producing (NP) cells previously described for Rous sarcoma virus (RSV) (Hanafusa *et al.*, 1964). The situations differed, however, in that morphologically distinguishable viral particles could be seen under the electron microscope (E.M.) in NP RSV hamster cells and that the presence of ALV group specific antigen in the NP cells, and of circulating antibody to ALV group antigen in the serum, could be demonstrated by the CFT (Sarma *et al.*, 1965). Recent trans-species rescue of the MSV genome from HT-1 cells by feline leukaemia virus (Sarma *et al.*, 1970) has suggested the possibility of a trans-species rescue of this genome from the HT-1 cells by a possible human leukaemia virus. HT-1 cells are clearly of great potential interest in research and we therefore considered it important to learn more about their cytology, cytochemistry, cytogenetics and ultrastructure.

### MATERIALS AND METHODS

### *Cell culture*

HT-1 cells were kindly provided by Dr. P. J. Fischinger of the National Cancer Institute, N.I.H., Bethesda. During our study the cells were maintained and propagated in Dulbecco's modified Eagle's medium (DMM) plus 10% calf serum. The medium also contained 200 units/ml. of penicillin, 200 $\mu$g./ml. of streptomycin, 5 $\mu$g./ml. of aureomycin and 25 $\mu$g./ml. of mycostatin.

### *Cytology and cytochemistry of HT-1 cells*

Since most HT-1 cells grew in suspension, the cells had to be spun down for the preparation of spreads on glass slides. After fixation the cells were stained using the following staining techniques (Hayhoe and Flemans, 1969): Leishman, May–Grunwald–Giemsa (MGG), acid phosphatase, periodic acid-Schiff (PAS), Sudan black and alkaline phosphatase. The acid phosphatase staining method is after Burton (1954) and the alkaline phosphatase is after Kaplow (1955).

Since some of the HT-1 cells settled on the glass surface during culture, they were trypsinized and seeded on coverslips in Leighton tubes. The cells which adhered to the glass were stained with MGG only.

### *Preparation of cells for karyotype analysis*

Colchicine was added to cultures of HT-1 cells and incubation at 37° C. was continued for 2 hours. Following incubation, the suspension of cells was centrifuged at 800 r.p.m. for 5 minutes, the growth medium discarded, and a hypotonic solution (0·0975M KCl) added to the remaining pellet of cells for 5 minutes. The cells were then fixed with Carnoy's fixative (1 part acetic acid and 3 parts methanol), air dried, and stained with Giemsa or Aceto Orcein. Only well dispersed chromosome spreads were studied.

Analyses of the chromosomes in HT-1 cells were grouped into three categories:

1. Cell population A consisted of all those HT-1 cells which originally grew in free suspension.
2. Cell population B was derived from those HT-1 cells which had originally tended to adhere to the glass surface. These cells were trypsinized, seeded in flasks, and later gave rise to further cells growing freely in suspension. These free cells constituted population B.
3. Cell population C consisted of a separate clone of cells derived from population B.

*Preparation of cells for electron microscope examination*

HT-1 cells were separated from culture medium by low speed centrifugation and then fixed as a suspension for 1 hour at room temperature in 1·5 % glutaraldehyde in 0·1M cacodylate-HCl buffer containing 1 % sucrose (pH 7·4). The cells were washed three times in 0·1M cacodylate-HCl buffer containing 7 % sucrose (pH 7·4) and then post-fixed for 2 hours at room temperature in 2 % osmium tetroxide in 0·1M cacodylate-HCl (pH 7·4). They were then dehydrated in one change each of 70 % and 90 % ethanol, 4 changes of absolute ethanol each of 5 minutes' duration, followed by two 15-minute changes of propylene oxide. Finally, the cells were embedded in Taab embedding resin. Thin sections were cut with glass knives on an L.K.B. ultratome III, doubly stained with uranyl acetate and lead citrate, and examined in an A.E.I. EM 6B electron microscope operated at 60 kV.

### RESULTS

*Cytology*

A very pleomorphic population of cells was revealed under the light microscope (Fig. 1–3). The majority of the cells had a single eccentric nucleus and showed great variation in total cell size and shape. Cells which were stained with MGG had a bluish (RNA) cytoplasm and a red (DNA) nucleus with blue (RNA) nucleoli (Jacobson and Webb, 1952). Many of the cells had cytoplasmic pseudopodia protruding to a variable extent (Fig. 1), and pleomorphic multinucleated cells of various size, often with unequal nuclei, were quite common. In some cells nuclear fragments could be seen. A few cells had cytoplasmic vacuoles. Cells which grew on the glass surface showed even greater cytoplasmic and nuclear pleomorphism (Fig. 2). There were giant multinucleated cells with nuclei of variable size and shape. It was not uncommon to find one or more phagocytosed cells in variable stages of digestion in the cytoplasm (Fig. 3). Certain nuclei appeared to have no chromatin aggregates, and stained light red with MGG in contrast to the bright red of the typical nuclei with condensed chromatin (Fig. 1). The nuclear membrane of the lightly stained nuclei always presented a smooth circular outline, with no margination of condensed chromatin, in contrast to the rather irregular outline of the nuclear membrane of most cells.

*Cytochemistry*

*Periodic acid-Schiff (PAS).*—Approximately 30–40 % of the cells showed a varying degree of positivity ranging from fine granules to small blocks. Although positivity was found throughout the cytoplasm, it tended to be particularly strong in pseudopodia. The glycogen nature of the PAS positivity was established by amylase digestion which completely abolished positivity (Fig. 4).

*Acid phosphatase.*—All cells showed some granular positivity ranging from moderately strong to strong (Fig. 5).

*Sudan black and alkaline phosphatase.*—Both these cytochemical reactions were entirely negative.

*Karyotype analysis*

*Population A.*—The HT-1 cells were found to be markedly aneuploid (Fig. 6). The number of chromosomes fluctuated around triploidy with structural rearrangements—the normal diploid being 44 (Fig. 7). Many cells contained a very striking and large telocentric marker chromosome (Ml) (Fig. 8). The telocentric chromosomes in the normal karyotype are much smaller (Fig. 7). Of the 26 chromosome spreads analysed, 16 (62 %) contained this large marker chromosome (Fig. 8), while in 6 cells (23 %) two telocentric marker chromosomes (Mla and Mlb), each about half the size of the Ml chromosome, were present (Fig. 9). In these six cells the large Ml chromosome was absent. One X chromosome was usually identifiable. A chromosome of similar size, in which one part of the arms was heavily condensed, was also present in most cells. This was probably a second X chromosome (Hampar and Ellison, 1961). Some cells which did not have this chromosome had the two smaller Ml chromosomes, which may suggest that these chromosomes might have been formed by a fracturing of a second X-like

FIG. 6.—A histogram showing the aneuploidy of the three cell populations.

chromosome (Fig. 9). Therefore, the large Ml and the smaller Mla and Mlb may be unrelated, and may in fact represent two cell lines. Many of the cells (54 %) also contained fragments or minute chromosomes which have been designated as M2 (Fig. 8), although probably not of identical origin. A detailed analysis of the HT-1 cells is outlined in Table I. The number of chromosomes ranged between 61 and 67 with a modal number of 64. No true triploid cells could be isolated. It is possible that this karyotype is not strictly typical of the tumour, since culture *in vitro* may lead to an adjustment of the cell karyotype, usually towards an increase in chromosome number, irrespective of the neoplastic properties of the cell (Harris, 1964). Colchicine treatment may also affect the karyotype (Harris, 1964).

*Cell population B.*—The number of chromosomes in this group ranged from 58–64 chromosomes (Table II, Fig. 6). The marker chromosome (Ml) could not be found in any of the 19 spreads studied. There were more cells with 2 X chromosomes in this cell line (Fig. 10). Minute chromosomes (M2) were found only in one cell.

*Cell population C.*—Cells for this clonal population did not have any large

TABLE I.—*Analysis of the Chromosome Numbers in 26 Metaphases from HT-1 Cells (Cell Population A)*

| | Normal male | | | | | | | | | | | | | | | | | | | | | | | | | | |
|---|---|---|---|---|---|---|---|---|---|---|---|---|---|---|---|---|---|---|---|---|---|---|---|---|---|---|---|
| Total number | 44 | 61 | 62 | 62 | 63 | 63 | 63 | 63 | 63 | 63 | 64 | 64 | 64 | 64 | 64 | 64 | 64 | 65 | 65 | 65 | 65 | 65 | 65 | 66 | 66 | 66 | 67 |
| *M1 | — | — | 1 | 1 | 1 | 1 | 1 | 1 | 1 | 2 | 1 | — | — | 1 | — | 2 | 1 | 1 | 1 | 1 | 2 | 2 | 2 | 2 | 1 | 1 | 1 |
| †Metacentric | — | 1 | — | 1 | 1 | 1 | — | — | 1 | 1 | 1 | 2 | 1 | 1 | 1 | 1 | 1 | 1 | 1 | 1 | — | 1 | — | 1 | — | 1 | — |
| Dicentric | — | — | — | — | — | — | — | — | 1 | — | — | — | — | — | — | — | 1 in 1–15 | — | — | — | — | — | — | — | — | — | — |
| X | 1 | 1 | 1 | 1 | 1 | 1 | 1 | 1 | — | — | 1 | 1 | — | 1 | 1 | — | 1 | 1 | 1 | 1 | 1 | — | 1 | — | 2 | 1 | 1 |
| 1–15 + Y | 31 | 37 | 36 | 38 | 39 | 38 | 40 | 37 | 41 | 37 | 38 | 38 | 41 | 43 | 39 | 38 | 40 | 38 | 37 | 39 | 38 | 41 | 39 | 35 | 40 | 38 | 38 |
| 16–19 | 8 | 16 | 14 | 12 | 12 | 13 | 10 | 14 | 10 | 15 | 16 | 16 | 13 | 12 | 15 | 14 | 13 | 15 | 15 | 13 | 15 | 13 | 14 | 19 | 13 | 15 | 17 |
| 20 | 2 | 2 | 5 | 4 | 4 | 5 | 3 | 5 | 3 | 4 | 2 | 2 | 3 | 2 | 4 | 5 | 3 | 4 | 5 | 4 | 5 | 4 | 5 | 4 | 3 | 4 | 5 |
| 21 | 2 | 4 | 4 | 5 | 5 | 4 | 4 | 4 | 4 | 4 | 4 | 4 | 4 | 4 | 4 | 4 | 4 | 5 | 4 | 5 | 4 | 4 | 4 | 3 | 4 | 5 | 4 |
| M2 | — | — | 1 | — | — | 1 | 1 | — | 1 | — | 1 | 1 | 2 | — | — | — | 1 | — | 1 | 1 pr | — | — | — | 2 | 2 | 1 | 1 |
| Deleted | — | — | — | — | — | — | — | — | Del. "20" | — | — | — | — | — | — | — | — | — | — | — | — | — | — | — | Del. "20" | — | — |

* Telocentric—if only one is present this is large (M1), when two are present they are both smaller (M1a and M1b) being about twice the size of the 16–19 group.

† Metacentric—this probably is a second X in a transformed state similar to that found in viral infections.

TABLE II.—*Analysis of the Chromosome Number in 19 Metaphases from HT-1 Cells Derived from Cells Adhering to the Glass Surface (Cell Population B)*

| | Normal male | | | | | | | | | | | | | | | | | | | |
|---|---|---|---|---|---|---|---|---|---|---|---|---|---|---|---|---|---|---|---|---|
| Total number | 44 | 58 | 58 | 60 | 60 | 60 | 61 | 61 | 62 | 62 | 62 | 62 | 62 | 63 | 63 | 63 | 63 | 64 | 64 | 64 |
| M1 | — | — | — | — | — | — | — | — | — | — | — | — | — | — | — | — | — | — | — | — |
| *Metacentric | — | — | — | 1 | — | 1 | 1 | 2 | 1 | 1 | 1 | 1 | — | 2 | — | — | 1 | — | — | — |
| Dicentric | — | 2† | 2 | — | — | — | — | — | — | — | — | — | — | — | — | — | — | — | — | — |
| X | 1 | 2 | 2 | 1 | 2 | 1 | 1 | 1 | 2 | 1 | 1 | 1 | 1 | 1 | 1 | 1 | — | 1 | 1 | 1 |
| 1–15 + Y | 31 | 36 | 38 | 39 | 36 | 36 | 42 | 37 | 41 | 38 | 42 | 38 | 40 | 42 | 40 | 39 | 39 | 40 | 40 | 39 |
| 16–19 | 8 | 12 | 10 | 13 | 13 | 14 | 12 | 15 | 12 | 14 | 12 | 16 | 13 | 12 | 14 | 15 | 14 | 15 | 15 | 15 |
| 20 | 2 | 2 | 2 | 2 | 2 | 3 | 2 | 2 | 2 | 3 | 2 | 2 | 4 | 2 | 4 | 3 | 4 | 3 | 4 | 4 |
| 21 | 2 | 4 | 4 | 4 | 4 | 5 | 4 | 4 | 4 | 5 | 4 | 4 | 4 | 4 | 4 | 5 | 5 | 5 | 4 | 5 |
| M2 | — | — | — | — | 2 prs. | — | — | — | — | — | — | — | — | — | — | — | — | — | — | — |
| Deletion | — | — | — | — | — | — | — | — | — | — | — | — | — | — | — | — | — | — | — | — |

* Metacentric probably a second X.

† One in 1–15 group.

marker chromosome (M1) or any minute chromosomes (M2). In spite of the cloning, the number of chromosomes fluctuated between 62–65 (Table III, Fig. 6 and 11), but there was a clear shift to a modal chromosome number of 65. There was an overall loss of chromosomes in group 20 with an increase in group 1–15.

*E.M. examination*

The tumour cells displayed a wide variation of size, shape, and internal structure, making it difficult to define any particular cell type (Fig. 12–16). No structures resembling complete C-type virus particles were seen in any of the cells examined, but a few cells contained particles showing some resemblance to virus, although it was difficult to distinguish these from the many pinocytotic vesicles found in these cells (Fig. 13, 17). The particles measured approximately 700 Å and some appeared to have radiating spikes. The ultrastructure of the cells showed many of the non-specific features found in exaggerated form in tumour cells; some of these will now be briefly described.

TABLE III.—*Analysis of Chromosome Number of* 13 *Metaphases from the Cloned Line of HT*-1 *Cell* (*Cell Population C*)

| | Normal male | | | | | | | | | | | | | |
|---|---|---|---|---|---|---|---|---|---|---|---|---|---|---|
| Total number | 44 | 62 | 63 | 63 | 64 | 64 | 64 | 64 | 65 | 65 | 65 | 65 | 65 | 65 |
| M1 | — | — | — | — | — | — | — | — | — | — | — | — | — | — |
| Metacentric | — | — | 1 | — | 1 | 1 | — | — | — | — | — | — | — | — |
| Dicentric | — | — | — | — | — | — | — | — | ?1 | — | — | — | — | — |
| X | 1 | 2 | 1 | 1 | 2 | 1 | 1 | 2 | 1 | 2 | 2 | 2 | 2 | 2 |
| 1–15 + Y | 31 | 39 | 39 | 41 | 41 | 40 | 45 | 39 | 41 | 42 | 42 | 39 | 41 | 41 |
| 16–19 | 8 | 15 | 16 | 15 | 13 | 16 | 12 | 17 | 16 | 15 | 12 | 18 | 16 | 16 |
| 20 | 2 | 1 | 1 | 1 | 2 | 1 | 2 | 2 | 2 | 1 | 1 | 2 | 1 | 1 |
| 21 | 2 | 5 | 5 | 5 | 5 | 5 | 4 | 4 | 4 | 5 | 8 | 4 | 5 | 5 |
| M2 | — | — | — | — | — | — | — | — | — | — | — | — | — | — |
| Deletion | — | — | — | — | — | — | — | — | — | — | — | — | — | — |

*Nucleus.*—Most cells contained a single nucleus but some contained more than one nuclear profile (Fig. 12, 15, 16). Although serial sections were not studied, it seems likely that most of these cells contained more than one nucleus rather than a single, lobulated nucleus since light microscopy revealed little evidence of nuclear lobulation. The nuclear outline was often relatively smooth, but occasionally deep invaginations of the nuclear membrane were seen. When cut tangentially these invaginations appeared as isolated membrane-enclosed areas of cytoplasm (Fig. 18 and 19). These pseudo-inclusions have been frequently described in tumour cells (Leduc and Wilson, 1959). The nucleolus was often multiple and frequently highly developed, containing both pars amorpha and nucleolonema in varying proportion (Fig. 12 and 18). Many nucleoli contained several distinguishable bodies with a central dense core (Fig. 12). These bodies resembled " microspherules " which have been observed in very active, more compact nucleoli such as those of cancer cells (Busch and Smetana, 1970). Some nuclei contained " dense bodies " (Fig. 19) which have been reported to occur frequently in nuclei of virus-induced tumours and human malignancies (Haguenau, 1969). Frequent " nuclear bodies " which have been reported in hamster cells transformed by RSV (Haguenau, 1969) were also seen (Fig. 12–15 and 18). Patches of interchromatinic granules, another non-specific feature of tumour cells, were also seen

(Fig. 12, 13, 18), although less frequently than dense bodies and nuclear bodies. Another observation of special interest is that in some cells there was a complete absence of chromatin aggregates (Fig. 14, 15, 16) usually present in the nucleus. Instead, the total nuclear material was arranged in a finely dispersed granulofilamentous pattern. In addition, these nuclei appeared to present an almost perfectly circular outline in striking contrast to the less regular outline of the other nuclei. A binucleated cell was observed in which one nucleus contained chromatin aggregates and was irregular in outline, while the other nucleus did not contain any chromatin aggregates and was circular (Fig. 19).

*Mitotic apparatus.*—Occasional mitoses and centrioles with radiating microtubules were observed (Fig. 19).

*Mitochondria.*—The cells displayed a very variable number of mitochondria. Some contained a large number (Fig. 12, 15, 16), while others appeared to contain only a few (Fig. 13). The mitochondria also varied widely in size and shape, but no consistent alteration was apparent.

*Lysosomes.*—Many cells contained abundant pleomorphic structures staining densely with uranyl acetate which presumably represented lysosomes. These lysosomes were randomly distributed throughout the cytoplasm except in the pseudopodia which usually contained very few organelles. This distribution of lysosomes corresponded closely with the distribution of acid phosphatase as seen cytochemically (Fig. 5). While the majority of cells showed this abundance of lysosomes, a few did not (Fig. 16).

*Other cytoplasmic organelles.* The Golgi apparatus was variably developed, being prominent in some cells and poorly developed in others. The endoplasmic reticulum was variably developed but never prominent (Fig. 12). Many cells contained free ribosomes scattered throughout the cytoplasm (Fig. 13), accounting for the basophilia seen in light microscopy. Frequent empty areas, often approximately circular in outline and not enclosed by a membrane, were observed in the cytoplasm (Fig. 14). These empty areas presumably contained material extracted during E.M. processing. The nature of this material was uncertain but PAS staining showed similar sized areas of glycogen suggesting that this material might be glycogen; alternatively, they might represent areas of lipid extracted during processing.

*Cell surface.*—The surface of many of these cells displayed numerous projections which were short and stubby or longer and more slender like microvilli. Some cells showed typical pseudopods largely devoid of organelles, but containing partially extracted glycogen as described above.

### DISCUSSION

In view of the past use of HT-1 cells in trans-species rescue of leukaemia viruses and the possible potential application in the search for a human leukaemia virus the present work was undertaken to define further the characteristics of these cells. Most HT-1 cells grow in fluid culture as individual cells or in clusters and can be propagated without any further trypsinization. Microscopic examination of the living cultures revealed a very pleomorphic cell population, heterogeneous in size and shape and with variation in size and shape of nuclei even within the same multinucleated cells. These characteristics and the presence of nuclear fragments of various sizes in the cytoplasm of some cells suggests the occurrence of abnormal cell divisions. There was also evidence of high phagocytic capacity (Fig. 3).

Of particular interest were those nuclei which appeared devoid of any chromatin aggregates in stained preparations (Fig. 1 and 2). The faint reddish staining of these nuclei suggested the presence of DNA in the dispersed state. Electron microscopic examination of similar nuclei supported this light microscope observation by confirming the absence of condensed chromatin within these nuclei (Fig. 14–16). In some multi-nucleated cells a nucleus without chromatin particles could be found next to one containing a normal chromatin pattern (Fig. 16).

---

### EXPLANATION OF PLATES

FIG. 1.—Three HT-1 cells which grew in free suspension showing variation in size as well as in nuclear number. It is of interest to note that one of the nuclei (arrow) does not contain any chromatin aggregates and even in this black and white print appears pale compared with the other nuclei. All three cells have prominent cytoplasmic pseudopodia. × 1600.

FIG. 2.—A giant HT-1 grown on the glass surface showing numerous nuclei of variable size and shape. The chromatin aggregates are absent in one of the nuclei (arrow). × 500.

FIG. 3.—A phagocytic binucleated cell showing two almost intact cells within its cytoplasm. × 590.

FIG. 4.—Cells stained by the PAS method showing a variable degree of positivity. Note that positivity tends to be concentrated in the pseudopodia. × 590.

FIG. 5.—Acid phosphatase staining indicates that all cells contain lysosomes. Note the great variation in the degree of positivity between individual cells. × 590.

FIG. 7.—The karyotype of a normal male Syrian hamster.

FIG. 8.—A typical karyotype of a population A cell showing M1, M2 and two X chromosomes. Note the increase in chromosome number and the evidence of chromosome rearrangement.

FIG. 9.—Karyotype of a population A cell showing M1a and M1b and one submetacentric X chromosome.

FIG. 10.—Karyotype of a population B cell. Note the absence of M1. Present is a metacentric X, the chromosome marked 1 may possibly be a Y.

FIG. 11.—Karyotype of a population B cell showing a metacentric and a submetacentric X. The chromosome marked 1 may possibly be a Y.

FIG. 12.—Showing two nuclear profiles, the larger containing a well developed nucleolus consisting of both pars amorpha and nucleolonema. The nucleolus contains " microspherules " (S inset). The nucleoplasma contains two " nuclear bodies " and a small patch of interchromatinic granules (g) (probably nuclear ribosomes). The cytoplasm shows lysosomes and mitochondria. Note the numerous cytoplasmic projections resembling microvilli. Strands of endoplastic reticulum can be seen. × 5175; inset × 14,850.

FIG. 13.—A mononuclear cell showing an irregularly shaped nucleus with margination of chromatin, one " nuclear body " (n) and patches of interchromatinic granules. The cytoplasm is relatively devoid of organelles, although several lysosomal structures are present. In one area of the cytoplasm (arrows) there are numerous particles which may represent incomplete virus. Many free ribosomes are scattered throughout the cytoplasm. × 12,800.

FIG. 14.—A mononuclear cell showing a nucleus devoid of chromatin aggregates and containing one " nuclear body ". The cytoplasm is rich in organelles and contains vacuoles (v) which are probably due to glycogen or lipids. × 4750.

FIG. 15.—A cell with 3 nuclei, all of which are devoid of chromatin aggregates. One nucleus contains a large nucleolus consisting entirely of pars amorpha. Another nucleus contains " nuclear bodies " (n). Note the perfectly rounded outline of the nuclear membrane. Cytoplasmic surface projections are well developed. × 6700.

FIG. 16.—Part of a binucleated cell showing one nucleus with an irregular outline containing chromatin aggregates, whilst the spherical nucleus is completely devoid of chromatin aggregates. A centriole with radiating microtubules can be clearly seen (C). The area marked with an arrow probably represents glycogen granules (gl.). × 19,600.

FIG. 17.—The lower magnification ( × 17,200) shows two vesicular structures (1 and 2), one with a myelin figure (2), surrounded by numerous small round particles. Inset are these two areas shown at higher magnification ( × 43,700) in which some of the particles are seen to have radiating spikes and measure approximately 700 Å in diameter (arrow).

FIG. 18.—The nucleus contains hypertrophied nucleoli, " nuclear bodies " (n), and patches of interchromatinic granules (g). In addition, a pseudoinclusion of cytoplasm is seen within the nucleus (I) which probably represents a deep invagination of the nuclear membrane cut in section. × 16,500.

FIG. 19.—Note several " dense bodies " (arrows) and a small pseudocytoplasmic inclusion (I) within the nucleus. × 10,800.

1

Karpas, Cawley, Tuckerman, Flemans and Hayhoe.

2

3

4

5

Karpas, Cawley, Tuckerman, Flemans and Hayhoe.

7

Karpas, Cawley, Tuckerman, Flemans and Hayhoe.

8

Karpas, Cawley, Tuckerman, Flemans and Hayhoe.

9

Karpas, Cawley, Tuckerman, Flemans and Hayhoe.

10

Karpas, Cawley, Tuckerman, Flemans and Hayhoe.

11

Karpas, Cawley, Tuckerman, Flemans and Hayhoe.

12

Karpas, Cawley, Tuckerman, Flemans and Hayhoe.

13

Karpas, Cawley, Tuckerman, Flemans and Hayhoe.

14

Karpas, Cawley, Tuckerman, Flemans and Hayhoe.

15

Karpas, Cawley, Tuckerman, Flemans and Hayhoe.

16

Karpas, Cawley, Tuckerman, Flemans and Hayhoe.

17

Karpas, Cawley, Tuckerman, Flemans and Hayhoe.

18

Karpas, Cawley, Tuckerman, Flemans and Hayhoe.

19

Karpas, Cawley, Tuckerman, Flemans and Hayhoe.

As far as the authors are aware, this type of nuclear structure does not seem to have been described previously in somatic cells of higher vertebrates either in connection with viral infection or otherwise. A possible explanation may be that in nuclei without chromatin the total genetic material was free DNA, not linked to a histone to form chromatin aggregates. Whether the presence within the affected cells of the viral genome plays some part in causing this unusual phenomenon to appear is clearly a question of considerable interest, requiring further investigation.

The acid phosphatase cytochemistry of the HT-1 cells showed a random distribution of enzyme activity (Fig. 5), (and therefore lysosomes) throughout the cytoplasm and rarely in pseudopodia; a distribution confirmed by electron microscopy (Fig. 12 and 15). In contrast, the PAS positivity (shown to be glycogen by amylase digestion) tended to be concentrated in the cytoplasm of pseudopodia (Fig. 4). E.M. study further revealed that the nuclei of some cells contained " dense bodies " (Fig. 19) as well as " nuclear bodies " (Fig. 12, 15, 18). " Nuclear bodies " have been described in RSV infected hamster cells (Haguenau, 1969). Since earlier study of HT-1 cells failed to demonstrate the presence of morphologically distinguishable viral particles in these cells (Huebner *et al.*, 1966; Valentine and Bader, 1968), the finding of the " nuclear and dense bodies " provides the best established morphological evidence at present available of the presence of the MSV genome in the cells, unless the particles which were found in the cytoplasm of some cells represented incomplete or immature virus (Fig. 13 and 17). These particles were certainly similar to the immature RSV particles described earlier by Dougherty *et al.* (1967).

The examination of the cell karyotype revealed the presence of a very large and striking telocentric marker chromosome in many cells (Fig. 8). The finding of this chromosome was of particular interest since the normal karyotype of the hamster does not carry any chromosome which resembles it; its origin could not be determined. In addition, all HT-1 cells were hyperdiploid with chromosome numbers around triploidy, but none was a true triploid. In spite of the instability in the number of chromosomes of the separate cloned line of cells studied, none developed the M1 or M2 chromosome, and the variation in chromosome number was smaller than in the original HT-1 cell population. It may be possible to learn whether the MSV genome is associated with the large marker chromosome by using the rescue technique. If the MSV genome could not be recovered from the cloned HT-1 line lacking this marker chromosome, close association between the viral genome and the chromosome would be implied.

The excellent technical assistance of Miss R. Britchford is gratefully acknowledged. Thanks are also due to Miss J. Thompson and Miss J. Casey for typing the manuscript.

This work was supported by a grant from the Leukaemia Research Fund.

J. C. was in receipt of an M.R.C. Junior Research Fellowship.

### REFERENCES

BURTON, J. F.—(1954) *J. Histochem. Cytochem.*, **2**, 88.

BUSCH, H. AND SMETANA, K.—(1970) in ' The Nucleolus '. New York (Academic Press), p. 481.

DOUGHERTY, R. M., DISEFANO, H. AND ROTH, F. K.—(1967) *Proc. natn. Acad. Sci., U.S.A.*, **58**, 808.

HAGUENAU, F.—(1969) ' Ultrastructure of the Cancer Cell '. In ' The Biological Basis of Medicine '. Edited by E. E. Bittar and N. Bittar. New York (Academic Press), Vol. 5, pp. 433–486.
HAMPAR, B. AND ELLISON, S. A.—(1961) *Nature, Lond.*, **192**, 145.
HANAFUSA, H., HANAFUSA, T. AND RUBIN, H.—(1964) *Proc. natn. Acad. Sci., U.S.A.*, **51**, 41.
HARRIS, M.—(1964) in ' Cell Culture and Somatic Variation '. New York (Holt, Rinehart and Winston), Chapter 4.
HARVEY, J. J.—(1964) *Nature, Lond.*, **204**, 1104.
HAYHOE, F. G. J. AND FLEMANS, R. J.—(1969) in ' An Atlas of Haematological Cytology '. London (Wolfe Medical Books), pp. 316–317.
HUEBNER, R. J., HARTLEY, J. W., ROWE, W. P., LANE, W. P. AND CAPPS, W. J.—(1966) *Proc. natn. Acad. Sci. U.S.A.*, **56**, 1164.
JACOBSON, W. AND WEBB, M.—(1952) *Expl Cell Res.*, **111**, 163.
KAPLOW, L. S.—(1955) *Blood*, **10**, 1023.
LEDUC, E. H. AND WILSON, J. W.—(1959) *J. biophys. biochem. Cytol.*, **6**, 427.
MOLONEY, J. B.—(1966) ' The Application of Studies in Murine Leukaemia to the Problems of Human Neoplasia '. In ' Some Recent Developments in Comparative Medicine '. Edited by T. W. Fiennes. London (Academic Press), pp. 251–257.
SARMA, P. S., VASS, W. AND HUEBNER, R. J.—(1965) *Proc. natn. Acad. Sci. U.S.A.*, **55**, 1435.
SARMA, P. S., LOG, T. AND HUEBNER, R. J.—(1970) *Proc. natn. Acad. Sci. U.S.A.*, **65**, 81.
VALENTINE, A. F. AND BADER, J. P.—(1968) *J. Virology*, **2**, 224.